# Non-reciprocal multifarious self-organization

**DOI:** 10.1038/s41565-022-01258-2

**Published:** 2022-12-12

**Authors:** Saeed Osat, Ramin Golestanian

**Affiliations:** 1 Max Planck Institute for Dynamics and Self-Organization (MPIDS), Göttingen, Germany; 2 Rudolf Peierls Centre for Theoretical Physics, University of Oxford, Oxford, UK

**Keywords:** Statistical physics, Molecular self-assembly

## Abstract

A hallmark of living systems is the ability to employ a common set of building blocks that can self-organize into a multitude of different structures. This capability can only be afforded in non-equilibrium conditions, as evident from the energy-consuming nature of the plethora of such dynamical processes. To achieve automated dynamical control of such self-assembled structures and transitions between them, we need to identify the fundamental aspects of non-equilibrium dynamics that can enable such processes. Here we identify programmable non-reciprocal interactions as a tool to achieve such functionalities. The design rule is composed of reciprocal interactions that lead to the equilibrium assembly of the different structures, through a process denoted as multifarious self-assembly, and non-reciprocal interactions that give rise to non-equilibrium dynamical transitions between the structures. The design of such self-organized shape-shifting structures can be implemented at different scales, from nucleic acids and peptides to proteins and colloids.

## Abstract (web summary)

Living systems employ a common set of building blocks that can self-organize into a multitude of different structures. Now, a model system based on programmed non-reciprocal interactions, which generically emerge in non-equilibrium systems with chemical activity, exhibits self-assembly pathways that feature temporal structures in the form of cyclic motifs.

## Main

In biological systems, small building blocks self-assemble into structures by taking advantage of the thermal agitations to find matching partners in the medium. To achieve such functionality in an artificial system, the challenge will be to design an interaction matrix between the building blocks so that they self-assemble into predefined units^1–5^. A remarkable feature in living systems is a notion of versatility of the building blocks, which allows self-assembly processes to make economical use of the same units under different conditions. Proposals for how to design systems with such a capability have been recently put forward, particularly in the context of the so-called multifarious assembly mixture model^6,7^. The main challenge in designing multifarious self-assembly is to encode the desired structures in terms of interactions between the components in the pool so that the structure will be memorized and retrieved when needed^8^. This goal is achieved up to a certain capacity by engineering the specific interactions between the components^2,6,7^, in analogy with the Hopfield neural network model^9,10^.

**Fig. 1:**
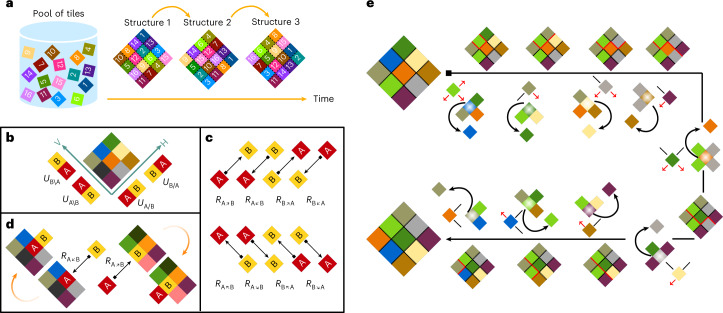
Broken action–reaction symmetry and multifarious self-organization model. **a**, Schematic of the realization of a sequence of programmed transitions between three different predefined target configurations. **b**, Specific reciprocal interactions between tiles A and B on a two-dimensional (H–V) lattice with specific binding energy *U*. **c**, Specific non-reciprocal interactions between A and B, and the corresponding interaction rates *R*. **d**, Non-reciprocity in the presence of a structure; A approaching B is different from B approaching A. **e**, Schematic of shape-shifting structures leveraging non-reciprocal interactions. Two desired structures are random permutations of 3 × 3 lattice configurations composed of nine distinct tiles. The non-reciprocal interactions induce transitions to the next structures. For arriving tiles, the red arrows and black lines show non-reciprocal and reciprocal interactions with the neighbouring tiles, respectively. The red boundary that emerges, grows and finally disappears represents the specific bonds that are lost due to the red arrows. This line shows how a small seed of the new structure emerges due to non-reciprocal interactions and eventually conforms to the full structure with the help of reciprocal interactions.

Living systems have also evolved to be able to choreograph the sequential formation of self-assembled structures in time from a common pool of tiles (Fig. 1a). Such self-organization, as exemplified in various stages of the cell cycle^11^, is not possible under equilibrium conditions. Therefore, to design such functionality, we need to identify a relevant non-equilibrium aspect of the process that is capable of driving time-sequenced stochastic dynamics. We propose programmable non-reciprocal interactions as a paradigm that provides sufficient conditions towards achieving this goal. Broken action–reaction symmetry has been recently explored in active matter in the context of non-equilibrium phoretic interactions between catalytically active colloids and enzymes^12^, and has shown to lead the formation of self-propelled active molecules that break time-reversal symmetry^13^, oscillating active complexes that break time-translation symmetry^14^, chiral bound states^15^ and active phase separation with specified stoichiometry^16,17^. Non-reciprocal interactions have been found to lead to rich physical phenomena involving various forms of spontaneous symmetry breaking in other related non-equilibrium contexts^18–24^, including early work in the context of asymmetric neural networks^25,26^. We note that a number of strategies have been recently pursued towards the experimental realization of shape-shifting soft-matter structures^27–30^.

Here we introduce the non-reciprocal multifarious self-organization model by incorporating non-reciprocal interactions into the equilibrium multifarious self-assembly model. We show that this non-equilibrium model is capable of inducing the shape-shifting property in the system in conjunction with multifarious self-assembly (Fig. 1a). We aim to find the best parameter space to realize this new shape-shifting regime and to characterize its properties. We show that shape shifters can be observed in a subset of the parameter space where multifarious assembly occurs. We find that the strength of the non-reciprocal interactions provides us with a control parameter to convert any selected multifarious self-assembly regime to the multifarious self-organization regime. We characterize the new shape-shifting behaviour by probing the frequency of shifts and the capacity and stability of cycle formation, as well as the entropy production that measures the degree of non-equilibrium activity in the self-organization process.

**Fig. 2:**
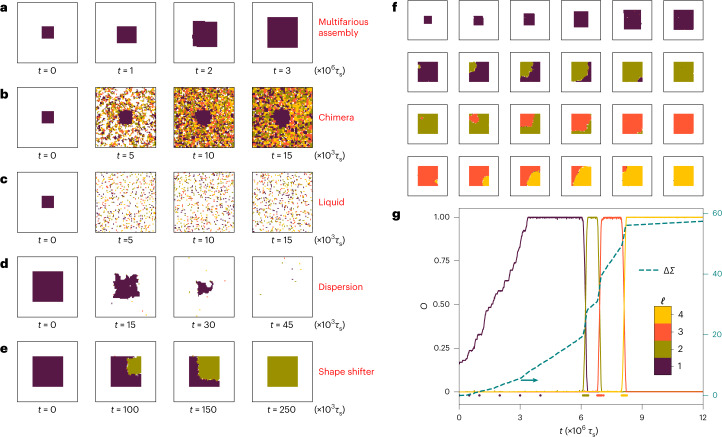
Shape-shifting self-organization. A structure is defined as a random arrangement of 1,600 tiles in a 40 × 40 square. Four desired structures are made by a random permutation of tiles and are encoded through reciprocal (equation (1)) and non-reciprocal (equation (4)) matrices. The shifting sequence is selected as {*S*^(1)^ → *S*^(2)^, *S*^(2)^ → *S*^(3)^, *S*^(3)^ → *S*^(4)^}. **a**–**e**, Possible outcomes of the simulation. **f**, More detailed transition path of the structures. Non-reciprocal interactions drive the non-equilibrium process that induces the transition between structures. Starting from the small initial seed of the first structure, the system retrieves the corresponding structure and then shifts to other structures in the sequence. **g**, Overlap of the system with each of the patterns as well as the corresponding entropy production are shown in the course of time evolution. The dots under the curves show the times at which snapshots have been recorded in **f**. The (seed size, *μ*, *ε*, *λ*) values are (16 × 16, −19, 11.0, 6) (**a**), (16 × 16, −10, 8.0, 5) (**b**), (16 × 16, 0, 3.0, 5) (**c**), (40 × 40, −10, 4.0, 0) (**d**), (40 × 40, −40, 25.0, 18) (**e**) and (16 × 16, −20, 11.5, 8) (**f**). Source data

Equilibrium self-assembly processes enable the spontaneous formation of predetermined structures from a pool of tiles. This can be achieved by designing directional interaction potentials that cover different encounters of two different tiles on a lattice (Fig. 1b). These interaction potentials are reciprocal, in the sense that any directional bonding incurs a given energy cost independently of how the tiles come to the binding arrangement. With such interactions, we can achieve self-assembly to the desired structure starting from an initial trigger—be it a small seed, enhanced concentration of some tiles and so on. However, this process will come to a stop once the self-assembled structure is achieved, and remain as such until the next trigger is introduced. Our aim here is to incorporate the ability of the system to autonomously switch from one self-assembled structure to the next. To this end, we define non-reciprocal interactions (Fig. 1c) by attributing different weights for every bond formation depending on which tile is being added and which tile is already a part of the cluster (Fig. 1d). Such non-reciprocal interactions give rise to a shape-shifting property amongst the ensemble of possible self-assembled structures from the pool of available tiles (Fig. 1a). We introduce and develop this paradigm in the context of a simple model and show how the interactions can be tuned such that many such transitions occur in a predefined sequence (Fig. 2).

To characterize the interactions between components A and B, one needs to define the interaction matrix or tensor (Methods). In the case of a square lattice, each component can specifically interact with four neighbours. To simplify the presentation, we introduce a handy notation that captures all the reciprocal and non-reciprocal interactions, which manifestly aids with the interpretation of the different interaction components. The lattice is described by horizontal (H) and vertical (V) directions, and a 45° rotation is applied to the lattice (\/), such we can denote the axes as ‘H’ / and ‘V’ \ (Fig. 1b). Then, specific horizontal (vertical) interactions between the tiles depending on the orientation can be captured by A/B (A\B), where A is the tile on the left (up) and B is the tile on the right (down). In the case of non-reciprocal interactions, the interactions can be classified by adding polarity to the orientations. For example, the horizontal interaction / breaks in two possibilities of ↗ and ↙, and the vertical interaction \ breaks in two possibilities of ↖ and ↘. All the configurations of the two tiles A and B and their resulting reciprocal and non-reciprocal interactions are summarized in Fig. 1b,c.

Programming equilibrium self-assembly boils down to designing interactions between tiles that are positioned next to each other in the desired structures. As shown in Fig. 1b, the directionality of the interactions is an important feature: depending on which faces of tiles A and B interact with each other (on contact), the interaction can be different. The interaction matrix *U* enumerates the list of all the specific combinations of pairs that are taken to be energetically favoured (Methods).

In our non-reciprocal implementation of the model, we need to complement the reciprocal specific bonds (or adjacency) with additional non-reciprocal variants. One can assume that beside every specific reciprocal bond characterizing which tile is preferentially positioned beside which other tile with what orientation, there is also non-reciprocal specific interaction specifying which tile is approaching the other tile (Fig. 1c). An illustration of this concept is shown in Fig. 1d. The situation in which the free tile B is approaching A—that is a part of a cluster—is characterized by the interaction (attraction or repulsion) acting on B from A, which is different from the case when the free tile A is approaching B and the relevant interaction will be characterized by what B does to A. Here the matrix *R* encodes the full classification of possible ways in which these approaches can be made advantageous (Methods).

An example of how such a transition between two different self-assembled structures can occur due to non-reciprocity is shown in Fig. 1e, where every step is initiated by the preferred directional non-reciprocal interaction between an incoming tile and existing cluster. There are two desired structures and the goal is to shift the first structure to the second one. This can be achieved by a design rule implemented by programming the *R* matrix. The design rule is built around events in which the tiles in the second pattern approach the tiles in the first pattern. This is done for each tile one by one, and respecting the geometry of the patterns. As an example, consider the first step shown in Fig. 1e, where the light green tile should replace the blue tile. This is achieved by introducing non-reciprocal interactions between the light green tile and the neighbours of the blue tile (shown as directed red arrows in this figure). Note that the incoming tile increases the energy of the system due to the removal of specific bonds between the outgoing tile and its neighbours. However, a low-energy configuration is recovered as soon as the second pattern starts to grow on top of the first pattern, similar to templating processes.

## Shape-shifting structure

The non-reciprocal multifarious self-organization model lives in a relatively high-dimensional parameter space, and therefore, the system can—in principle—exhibit a plethora of different behaviours. Figure 2a–e shows the possible outcomes of the model with different input parameters for energy *ε*, chemical potential *μ* and non-reciprocal interaction strength *λ* for *m* = 4 structures. A typical successful self-assembly of a desired structure starting from a small initial seed, representing the so-called ‘multifarious assembly’ process, is shown in Fig. 2a. The promiscuous tendency of interacting tiles can potentially result in the growth of a ‘chimera’ from an initial seed and hence leading to unfaithful self-assembly (Fig. 2b). The ‘liquid’ regime is characterized by the presence of tiles in the system without many specific interactions (Fig. 2c). The relatively small size of the largest specifically connected cluster of the system and the continuous change in the arrangements of tiles lead to a liquid-like structure. When the initial seed is not stable and dissolves, a dilute ‘dispersion’ can result (Fig. 2d). The instability of the seed can occur either due to a competition between energy and entropy of mixing (as represented by the chemical potential or density) or a strong non-equilibrium activity due to non-reciprocal interactions (Supplementary Information). Finally, a newly observed ‘shape-shifter’ regime is shown in Fig. 2e, in which an appropriate selection of parameters enables the system to switch an initial pattern to the next by exploiting non-reciprocal interactions.

The details of the shape-shifting behaviour is shown in more detail in Fig. 2f. Four different structures are stored in the mixture using appropriate choices for the *U*^r^ matrix elements, and the corresponding full sequence with a length of three, namely, {*S*^(1)^ → *S*^(2)^, *S*^(2)^ → *S*^(3)^, *S*^(3)^ → *S*^(4)^}, has been implemented using appropriate choices for the *R*^nr^ matrix elements (Methods). The simulation starts with introducing a small seed selected from the first structure, which grows and self-assembles to *S*^(1)^. Subsequently, the non-reciprocal interactions come into effect and cause *S*^(1)^ to switch to *S*^(2)^, then proceed to *S*^(3)^ and later to *S*^(4)^. To quantify this feature, Fig. 2g shows the overlap of the observed configuration with *S*^(*i*)^ (Methods and Supplementary Information). This shifting behaviour emerges for a sufficiently large *λ*.

The non-equilibrium characteristics of the non-reciprocal multifarious self-organization model can be probed by measuring entropy production^31–33^. We define entropy production (in units of *k*_B_, the Boltzmann constant) in our model as the sum of the logarithm of the ratio of probabilities for direct and reverse moves along the path of the dynamics: ${{\Delta }}{{\varSigma }}=\sum \log \frac{{P}_{\to }}{{P}_{\leftarrow }}$, where *P*_→_ and *P*_←_ represent the probabilities of forward and reverse moves, respectively. The example in Fig. 2g shows that Δ*Σ* is an informative readout of the degree of non-equilibrium activity in the course of the time evolution of the system.

**Fig. 3:**
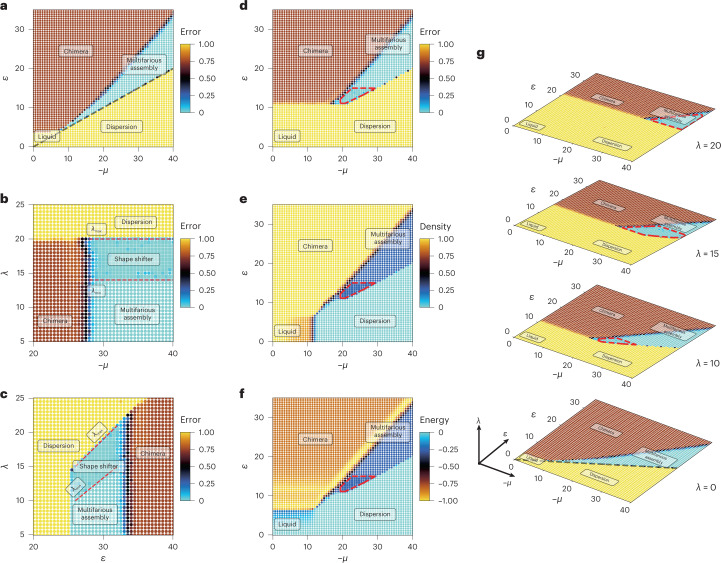
Emergence of shape-shifter behaviour within the multifarious assembly regime. Four different structures of size 40 × 40 are encoded through reciprocal (equation (1)) and non-reciprocal (equation (4)) interactions as a sequence with a length of three. Each point is an average of error over five (ten) independent realizations of the system measured after 4 × 10^6^*τ*_s_ time steps. Different forms of self-assembly are obtained starting from the first structure as an initial seed. **a**, Self-assembly error for the equilibrium case (*λ* = 0). The error delineates different modes of self-assembly. The grey dashed line shows the stability limit of the initial structure corresponding to the 2*ε* > −*μ* condition. **b**, Systematic scan of the (*λ*, *μ*) parameter space for fixed *ε* = 21 and −*μ* ∈ [20, 40]. The square markers correspond to the points that undergo at least one shift. Increasing *λ* beyond the threshold value (*λ*_min_) induces shifting between the structures and converts the multifarious assembly regime to the shape-shifting regime (square markers). A strong non-equilibrium drive for *λ* > *λ*_max_ makes the initial seed unstable and results in a dilute dispersion. **c**, Same data as **b**, but for the (*λ*, *ε*) parameter space for fixed *μ* = −30 and *ε* ∈ [10, 30]. The red dashed lines are a guide for the eyes through the limiting values of *λ*. **d**, Average self-assembly error for *λ* = 10. The red dashed line is the convex hall of points that corresponds to an error of <0.05 and has undergone at least one shift. **e**,**f**, Same data as **d**, but for density and energy at the final configuration. The boundary between the phases can be delineated using three quantities: error, density and energy (Extended Data Figs. 1–4). **g**, Emergence of a shape-shifting domain in the multifarious assembly region of the phase diagram. Source data

**Extended Data Fig. 1:**
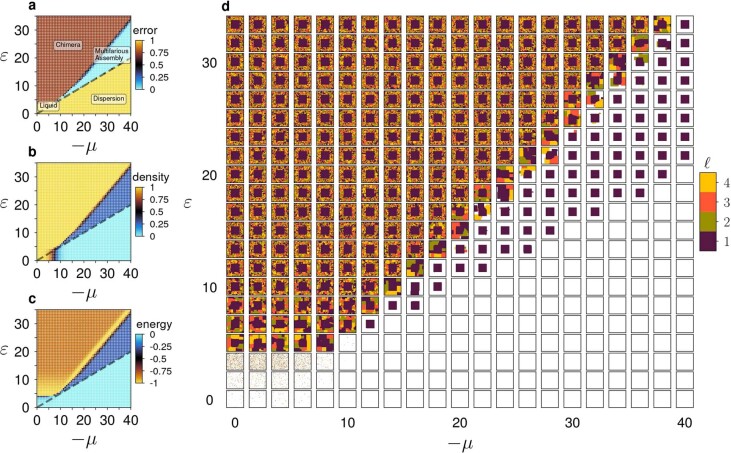
Multifarious assembly. Four different structures of size 40 × 40 are encoded through reciprocal interactions. In panel **a** each point is an average of error over 5 independent realizations of the system measured after 4 × 10^6^*τ*_*s*_ time steps. Different forms of self-assembly are obtained starting from the first structure as an initial seed. Grey dashed line shows the stability limit of the initial structure corresponding to the 2*ε* > − *μ* condition. Panels **b** and **c** are the same as **a** but for density and energy at the final configuration. The boundary between the phases can be delineated using the three quantities of error, density, and energy. **d**, Sample snapshots of the system at the end of simulations for pairs of (*μ*, *ε*) values. Note that the boundary between multifarious assembly and chimera regimes as well as the boundary between liquid and chimera regimes are filled with low energy chimeras in the form of homogeneously-nucleated structures.

We have systematically explored the parameter space of the non-reciprocal multifarious self-organization model, to uncover the conditions for obtaining the desired behaviour. The behaviour of the system at equilibrium (*λ* = 0) is described in Fig. 3a, where the error of self-assembly is used to identify the different regimes, namely, chimera, liquid, dispersion and multifarious self-assembly^7^. We note that error by itself may not be sufficient to determine the phase of the final configuration, and therefore, it will be important to simultaneously monitor the density, and sometimes, the energy (Extended Data Fig. 1 and Supplementary Information).

To explore the effect of non-reciprocal interactions, we plot the error for different values of (*λ*, *μ*) (Fig. 3b) for fixed *ε*. We observe that for small values of *λ*, the self-assembled structures are stable, whereas increasing *λ* beyond a threshold *λ*_min_ introduces the shape-shifting behaviour. As expected, the shifts only happen in the multifarious assembly region. When *λ* is larger than a second threshold *λ*_max_, the seed is destabilized and a dispersion is observed. A similar behaviour is observed for fixed *μ* in the space of (*λ*, *ε*) (Fig. 3c).

To shed more light on how the shape-shifter domain can be obtained, we present the diagram describing the behaviour of the system in the space of (*μ*, *ε*) for different values of *λ* (Fig. 3d–g). In the equilibrium multifarious assembly model (Extended Data Fig. 1), the self-assembly regime is sandwiched between the dispersion of equilibrium instability of the initial seed and chimera regime. Both liquid and multifarious assembly regimes are separated from the chimera regime by a band of homogeneously nucleated chimeras marked by the lowest energy (Extended Data Fig. 1c).

**Extended Data Fig. 2:**
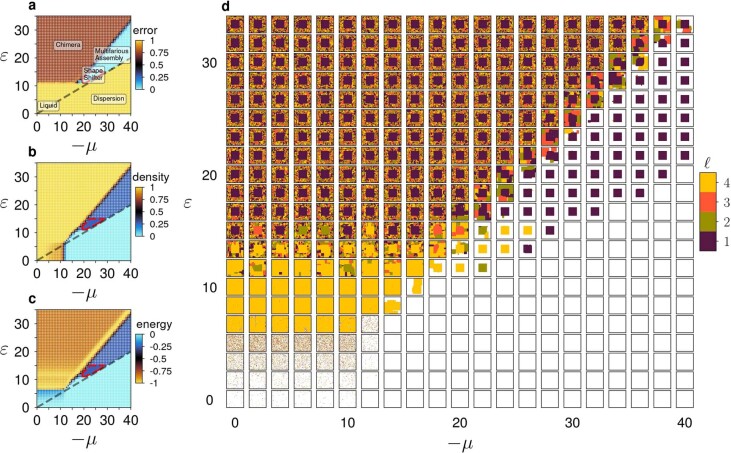
Emergence of shape shifter within the multifarious assembly regime: weak non-reciprocal drive. Four different structures of size 40 × 40 are encoded through reciprocal and non-reciprocal interactions as a sequence of length three. Strength of non-reciprocal interactions is set to *λ* = 10. In panel **a** each point is an average of error over 5 independent realizations of the system measured after 4 × 10^6^*τ*_*s*_ time steps. Different forms of self-assembly are obtained starting from the first structure as the initial seed. The square markers correspond to the points that undergo at least one shift. Shifts occur in the range of *ε* that respect the bounds $\frac{2}{3}\varepsilon \approx {\lambda }_{\min }\le \lambda \le {\lambda }_{\max }\approx \varepsilon$. Increasing *λ* beyond the threshold value ${\lambda }_{\min }$ induces shifting between the structures and converts the multifarious assembly regime to a shape-shifting regime (square markers). Strong non-reciprocal drive for $\lambda > {\lambda }_{\max }$ makes the initial seed unstable and results in a dilute dispersion. Grey dashed line shows the stability limit of the initial structure corresponding to the 2*ε* > − *μ* condition. The red dashed line is the convex hull of the points that correspond to error < 0.05 and have undergone at least one shift. Panels **b** and **c** are the same as **a** but for density and energy at the final configuration. The boundary between the states can be delineated using the three quantities error, density, and energy. **d**, Sample snapshots of the system at the end of the simulations for pairs of (*μ*, *ε*) values. Note that the liquid regime is extended and the liquid-chimera boundary is placed at higher *ε*.

**Extended Data Fig. 3:**
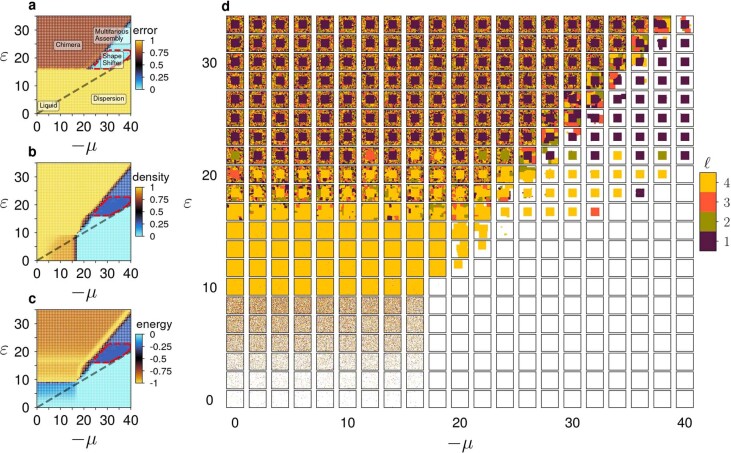
Emergence of shape shifter within the multifarious assembly regime: moderate non-reciprocal drive. Four different structures of size 40 × 40 are encoded through reciprocal and non-reciprocal interactions as a sequence of length three. Strength of non-reciprocal interactions is set to *λ* = 15. In panel **a** each point is an average of error over 5 independent realizations of the system measured after 4 × 10^6^*τ*_*s*_ time steps. Different forms of self-assembly are obtained starting from the first structure as the initial seed. The square markers correspond to the points that undergo at least one shift. Shifts occur in the range of *ε* that respect the bounds $\frac{2}{3}\varepsilon \approx {\lambda }_{\min }\le \lambda \le {\lambda }_{\max }\approx \varepsilon$. Increasing *λ* beyond the threshold value ${\lambda }_{\min }$ induces shifting between the structures and converts the multifarious assembly regime to a shape-shifting regime (square markers). Strong non-reciprocal drive for $\lambda > {\lambda }_{\max }$ makes the initial seed unstable and results in a dilute dispersion. Grey dashed line shows the stability limit of the initial structure corresponding to the 2*ε* > − *μ* condition. The red dashed line is the convex hull of the points that correspond to error < 0.05 and have undergone at least one shift. Panels **b** and **c** are the same as **a** but for density and energy at the final configuration. The boundary between the states can be delineated using the three quantities error, density, and energy. **d**, Sample snapshots of the system at the end of the simulations for pairs of (*μ*, *ε*) values. Note that the liquid regime is extended and the liquid-chimera boundary is placed at higher *ε*.

**Extended Data Fig. 4:**
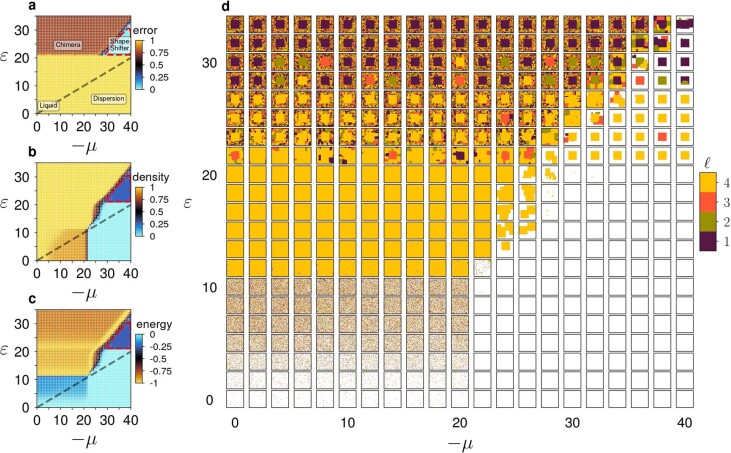
Emergence of shape shifter within the multifarious assembly regime: strong non- reciprocal drive. Four different structures of size 40 × 40 are encoded through reciprocal and non-reciprocal interactions as a sequence of length three. Strength of non-reciprocal interactions is set to *λ* = 20. In panel **a** each point is an average of error over 5 independent realizations of the system measured after 4 × 10^6^*τ*_*s*_ time steps. Different forms of self-assembly are obtained starting from the first structure as the initial seed. The square markers correspond to the points that undergo at least one shift. Shifts occur in the range of *ε* that respect the bounds $\frac{2}{3}\varepsilon \approx {\lambda }_{\min }\le \lambda \le {\lambda }_{\max }\approx \varepsilon$. Increasing *λ* beyond the threshold value ${\lambda }_{\min }$ induces shifting between the structures and converts the multifarious assembly regime to a shape-shifting regime (square markers). Strong non-reciprocal drive for $\lambda > {\lambda }_{\max }$ makes the initial seed unstable and results in a dilute dispersion. Grey dashed line shows the stability limit of the initial structure corresponding to the 2*ε* > − *μ* condition. The red dashed line is the convex hull of the points that correspond to error < 0.05 and have undergone at least one shift. Panels **b** and **c** are the same as **a** but for density and energy at the final configuration. The boundary between the states can be delineated using the three quantities error, density, and energy. **d**, Sample snapshots of the system at the end of the simulations for pairs of (*μ*, *ε*) values. Note that the liquid regime is extended and the liquid-chimera boundary is placed at higher *ε*.

With increasing *λ*, the regions in the equilibrium diagram change differently. Figure 3d–f corresponds to *λ* = 10, and are based on probing the error, density and energy (Extended Data Fig. 2). As expected, for sufficiently large *λ*, the multifarious assembly domain shrinks, as the part of it where *λ* ≥ *λ*_max_ ≈ *ε* becomes unstable for the initial seed. On the other hand, the parts in the remainder of the multifarious assembly domain that satisfy $\lambda \ge {\lambda }_{\min }\approx \frac{2}{3}\varepsilon$ can now accommodate a new shape-shifter region (Fig. 3d, red dashed line). Increasing *λ* further shrinks the domain and this trend continues, as shown in Fig. 3g (Extended Data Figs. 2–4).

The border between the liquid and chimeras also shifts towards higher energies with increasing *λ*. Although the liquid region extends, the former liquid–chimera border partially changes to liquid and partially to a new chimera where any possible bond with non-reciprocal interactions undergoes a shift until no more shift is available (Extended Data Fig. 2d, full yellow snapshots). The value of *λ* = 10 is not sufficiently large to induce changes in the diagram above $\varepsilon =\frac{3}{2}\lambda$. The same behaviour is shown in Extended Data Figs. 3 and 4 for *λ* = 15 and *λ* = 20, respectively.

## Frequency of shifts

**Fig. 4:**
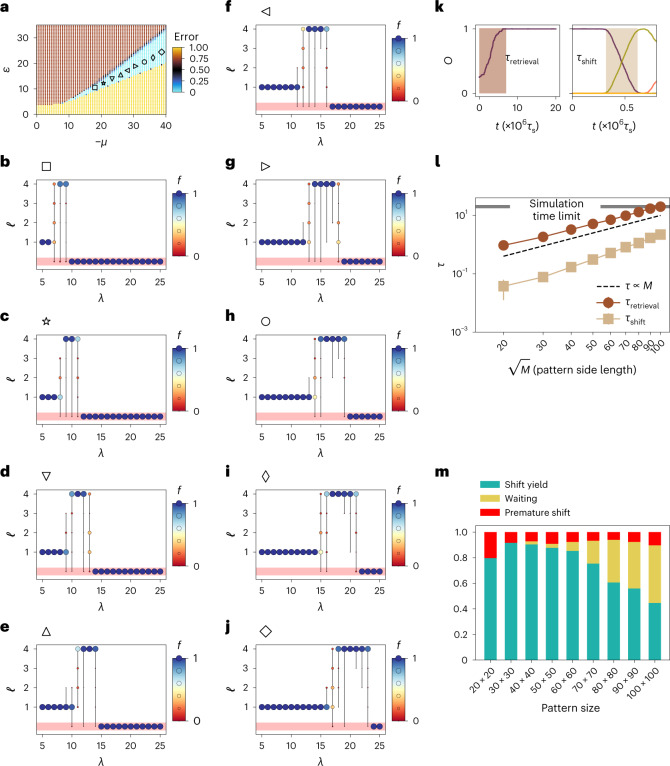
Frequency of shifts and scaling. **a**, Frequency of shifts is characterized for a number of different points in the multifarious assembly regime, as labelled by different markers. The setup corresponds to four desired structures of size 40 × 40, making a sequence with a length of three. **b**, Frequency of shifts for the square marker. Each multibulleted bar corresponds to an average of 100 independent realizations. A bullet appears at height *ℓ* if at least one of the realizations ended in the corresponding structure *S*^(*ℓ*)^. The size and colour of the bullets are related to the frequency or fraction of the realizations that stopped at the corresponding structure at the end of the simulation. The red band corresponds to any erroneous configuration; configurations with error of >0.1. **c**–**j**, Same data as **b**, but for different (*ε*, *μ*) values as marked in **a**. **k**, Definition of timescales of retrieval and shift. **l**, Scaling of both timescales (in units of 10^6^*τ*_s_ steps) with pattern side length. **m**, Yield of correct shift, premature shifts and shifts waiting in the queue. For the timescale analysis, we have used (seed size, *μ*, *ε*, *λ*): for retrieval, $(\sqrt{M}/2\times \sqrt{M}/2,20,11.5,0)$; for shifts, $(\sqrt{M}\times \sqrt{M},20,11.5,8)$. Source data

Let us now characterize the robustness of the shifting behaviour. We focus on the region of interest in the space of (*μ*, *ε*) (Fig. 4a). At each point marked in the multifarious assembly region (Fig. 4a), we change *λ* ∈ [5, 25]. To probe the quality of shifts, we define the frequency *f* as the fraction of independent realizations of the system that terminated the shifting at one of the configurations along the designed sequence, after a finite simulation time of 4 × 10^6^*τ*_s_ steps, with *τ*_s_ being one lattice sweep.

The final configuration can be any of the four states *S*^(1)^, *S*^(2)^, *S*^(3)^ and *S*^(4)^ for any valid shift, or anything else including the liquid, chimera and dispersion. From the functionality point of view, anything else is an erroneous structure and is not of any use. Figure 4b–j shows the frequency at different points in the multifarious assembly region. The red band captures erroneous structures. As observed in the figure, for each marker, there is a functional range of *λ* values that can induce the desired shifting between the structures (Supplementary Information).

## Timescales and yield

The mechanisms of self-assembly or retrieval of the desired structure and shifts are different. This can be seen from the timescale in which these two processes occur. The time that the initial seed with a size of 25% of the pattern, grows and reaches 95% is defined as *τ*_retrieval_. Also, *τ*_shift_ is defined as the time that it takes for the overlap to go from *O*_*i*_ = 0.95 to *O*_*i*+1_ = 0.95, where *i* and *i* + 1 are two consecutive structures in a sequence (Fig. 4k). The scaling of the two timescales with a side length of pattern is shown in Fig. 4l.

**Extended Data Fig. 5:**
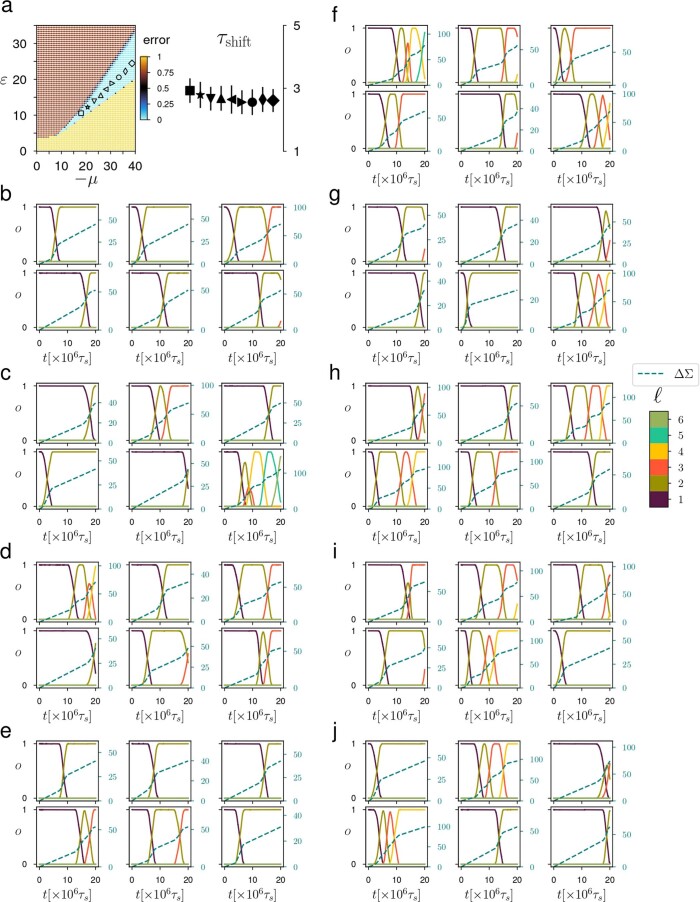
100 × 100 patterns. We stored 6 patterns of size 100 × 100 in a sequence of length 5. For different values of (*μ*, *ε*, *λ*) shown by different markers in **a** (left) we simulated 50 different realizations for ${{{\mathcal{T}}}}=20\times 1{0}^{6}{\tau }_{s}$ steps. Each panel from **b** to **j** shows 6 different realizations. Even for these large patterns, clear shift events are observed. *τ*_shift_ is calculated for each realization and the average value is shown in **a** (right), in units of 10^6^*τ*_*s*_ steps. Remarkably, *τ*_shift_ is almost independent of the choice of (*μ*, *ε*).

In sequence implementation, not all the shifts are successfully completed. Premature shifting refers to the realization of the shifting process from *S*^(*ℓ*)^ to the next structure *S*^(*ℓ*+1)^ before the completion of the self-assembly of the current structure *S*^(*ℓ*)^ in the shifting sequence {…, *S*^(*ℓ*)^ → *S*^(*ℓ*+1)^,…}. This can happen mainly due to the fact that the current structure grows in the boundary whereas the next shift takes place in the area of the current structure. The probabilities of these two processes are proportional to the boundary and area, respectively. Although unavoidable, we show that these premature shifts are only a fraction of all the possible shifts. For the same numerical results of timescale analysis, we looked at the number of correct shifts, premature shifts and waiting shifts. As shown in Fig. 4m, even for very large patterns, a large fraction of shifts are correctly completed. Note that as expected and as the timescale predicts, a fraction of waiting shifts increases as the pattern size increases. However, the overall behaviour of the system remains the same as the system size increases (Extended Data Fig. 5 shows a 100 × 100 realization of the system). We have also studied the interplay between the capacity of the system and the non-reciprocal coupling, cycle-forming sequences and their timescales, as well as basins of attraction of the different states in short cycles (Supplementary Information).

## Brownian dynamics simulation

**Extended Data Fig. 6:**
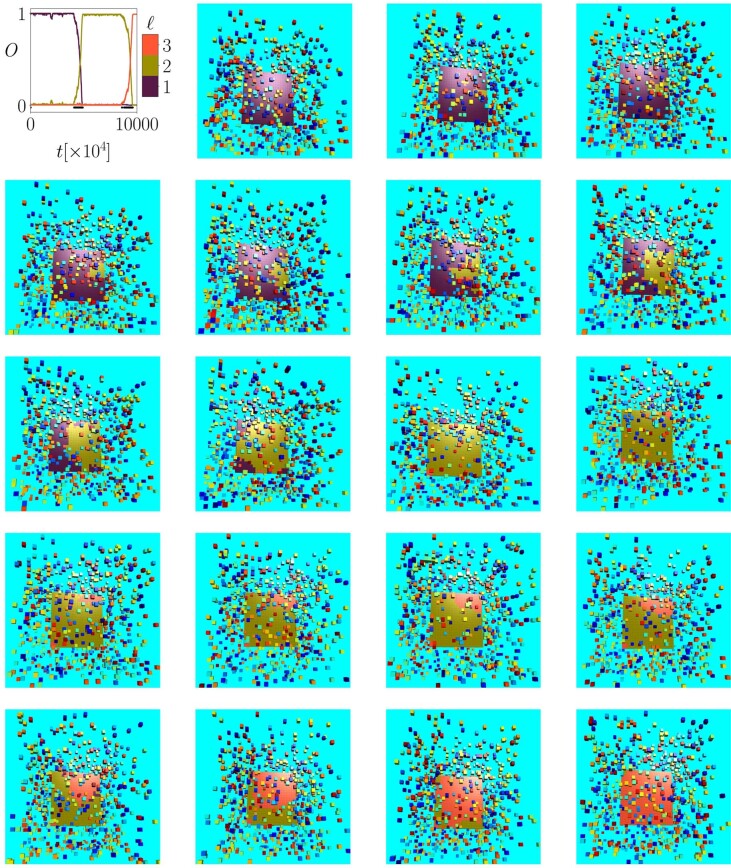
Brownian dynamics simulation. Three patterns of size 20 × 20 are stored as a sequence of length two; *S*^(1)^ → *S*^(2)^ → *S*^(3)^. The first pattern is placed in the middle of the box as an initial seed. Beside the initial seed the box contains two more copies of each tile; in total 1200 tiles are used in the simulations. Top left corner shows the evolution of the overlap of the part of the system that contains the initial seed with the desired patterns. Snapshots of the system are taken from the time steps marked with black dots. Other simulations parameters are (*ε*, *λ*) = (11.5, 8.0).

To help guide a practical implementation of the ideas presented here in practice, we have performed a three-dimensional Brownian dynamics simulation in which the self-organization of tiles is initiated with a seed that is placed in the middle of the box (Methods and Supplementary Information). Although a lattice implementation of square tiles is used to directly enable a comparison with the Monte Carlo simulation, we expect to obtain similar results for a continuum implementation of the model as well. Our observations confirm the main findings presented above, as shown by the shape-shifting time evolution presented in Extended Data Fig. 6 (Supplementary Fig. 20 shows another example in which premature shifting is observed).

## Conclusions

We have introduced the non-reciprocal multifarious self-organization model, which is capable of both retrieval of stored structures and inducing choreographed transitions between them, hence realizing shape-shifting structures. Through extensive simulations and systematic scanning of the parameter space of the model, we have demonstrated the feasibility of the shape-shifter design strategy that is triggered by non-reciprocal interactions. Therefore, we have identified programmable non-reciprocal interactions as a non-equilibrium paradigm using which the automated dynamical control of self-assembled structures and transitions between them can be realized. We have shown that the underlying mechanism of retrieval and shifts take place at different timescales. Moreover, we have demonstrated how our strategy can also find application in avoiding kinetics traps of chimera formation, which is a notorious problem of equilibrium self-assembly (Supplementary Information provides a detailed discussion).

There have been a number of studies concerning how specific interactions can be designed in colloidal systems such that desired self-assembly routes can be experimentally realized^2,34,35^, although these ideas have been explored in the context of protein complexes as well^7^. On the other hand, catalytically active colloids and enzymes with effective phoretic interactions have been shown to exhibit non-reciprocal interactions^13–17^, which are required for the shape-shifting behaviour to emerge. Therefore, using colloidal particles and enzymes as building blocks appears to provide a promising and natural route to the realization of the proposals presented here.

There are subtleties associated with the practical implementation of the non-reciprocal multifarious self-organization model. One aspect is a reduction in the capacity of the system compared with its equilibrium counterpart, which can be remedied by optimizing the trade-off between the heterogeneity of the target structures and sparse usage of the pool, as suggested elsewhere^7^. The premature shifting between the structures in the queue (mostly for strong non-reciprocal interactions) is another challenge, as discussed above. In connection to this issue, we note that the asymmetric Hopfield network model has been shown to have similar limitations, including chaotic behaviour in some circumstances^9,36,37^. In this context, it has been shown that a slow response of the spins can make it possible to circumvent this problem and even lead to the emergence of additional features^25,26^. This brings the idea that time-dependent specific interactions^38,39^ can provide a potential solution to help prevent premature shifts and avoid chaotic behaviour in the self-organization process in our model; moreover, it may even lead to new phenomena^34,39^. However, this comes with the complexity of the addition of a timescale to the parameter space of the design. These issues will be addressed in our follow-up studies using an appropriate non-equilibrium formalism.

## Methods

### Reciprocal interactions

We consider *m* desired structures with each being a random permutation of *M* components or tiles. For simplicity, let us arrange the first *q* structures in a queue that we label as *S*^(1)^ to *S*^(*q*)^, which defines the shifting sequence. The goal is to define an interaction matrix that enables the self-assembly of each target structure and the realization of the shifting sequence. We assume that each pair of neighbouring tiles in the desired structures has a specific interaction and define these reciprocal interactions imposed by structures as

1 $${U}_{A\square B}^{{{{\rm{r}}}}}=\left\{\begin{array}{ll}-\varepsilon ,\quad &{{{\rm{if}}}}A\square B\in {{{{\mathcal{I}}}}}^{{{{\rm{r}}}}},\\ 0,\quad &{{{\rm{otherwise}}}},\end{array}\right.$$

in units of the thermal energy *k*_B_*T*, where □ ∈ {\, /} represents a specific reciprocal interaction (Fig. 1b). Moreover, ${{{{\mathcal{I}}}}}^{{{{\rm{r}}}}}\equiv {I}^{{{{\rm{r}}}}}({S}^{(1)})\cup {I}^{{{{\rm{r}}}}}({S}^{(2)})\cup \cdots \cup {I}^{{{{\rm{r}}}}}({S}^{(m)})$ is the set of all the specific interactions between the tiles imposed by *m* desired structures, where *I*^r^(*S*^(*ℓ*)^) is the set of all the specific interactions in the structure *ℓ*, namely,

2 $${I}^{{{{\rm{r}}}}}({S}^{(\ell )})=\mathop{\bigcup}\limits_{\begin{array}{c}\left\langle \alpha ,\beta \right\rangle \end{array}}\,{S}_{\alpha }^{(\ell )}\square {S}_{\beta }^{(\ell )},$$

where *α* and *β* are the representatives of lattice coordinates (*i*, *j*) running over the nearest neighbours, and □ ∈ {\, /}. To describe the configuration space of the system, we can define a Potts configuration variable *σ*_*α*_ = 0, 1, 2,…*M*, with *σ*_*α*_ = 0 representing an empty slot and the others describing the corresponding tile species. Using the interaction potential and configuration variables, one can define a (classical generalized) Hamiltonian for the system as

3 $${{{\mathcal{H}}}}=\mathop{\sum}\limits_{\left\langle \alpha ,\beta \right\rangle }{U}_{{\sigma }_{\alpha }\square {\sigma }_{\beta }}^{{{{\rm{r}}}}}-\mu \,n,$$

where *μ* is the chemical potential of the tiles (assumed to be the same for all the species), $n={\sum }_{\alpha }\left(1-{\delta }_{0,{\sigma }_{\alpha }}\right)$ representing the total number of tiles in every given configuration and □ ∈ {\, /}. As usual, the chemical potential controls the average density of tiles in the system.

The reciprocal interaction *U* asserts that two components specifically interact if the interaction is favoured at least by one of the structures^6,7^. This simple interaction rule makes multifarious self-assembly model an associative memory capable of retrieving stored structures starting from an initial seed or any similar trigger. With an appropriate tuning of the model parameters (energy scale *ε*, number of components *M*, number of memorized patterns *m* and chemical potential *μ*), one can achieve an equilibrium self-assembly machine reminiscent of the Hopfield neural network^6,33,40^.

### Non-reciprocal interactions

The addition of a non-reciprocal flavour to specific interactions turns the equilibrium multifarious self-assembly model into the non-equilibrium multifarious self-organization model with a new shape-shifting property. Inspired by recent diverse physical models with non-reciprocal interactions^18–26^, we introduce non-reciprocal interactions between the tiles as follows. We define

4 $${R}_{A\blacksquare{B}}^{{{{\rm{nr}}}}}=\left\{\begin{array}{ll}\lambda ,\quad &{{{\rm{if}}}}\,A\,\blacksquare{B}\in {{{{\mathcal{I}}}}}^{{{{\rm{nr}}}}},\\ 0,\quad &{{{\rm{otherwise}}}},\end{array}\right.$$

where ■ ∈ {↘, ↖, ↗, ↙} represents all the possible specific non-reciprocal interactions (Fig. 1c). The set of all such interactions between the tiles needed to realize the shifting sequence {*S*^(1)^ → *S*^(2)^, *S*^(2)^ → *S*^(3)^…*S*^(*q*−1)^ → *S*^(*q*)^} is denoted by ${{{{\mathcal{I}}}}}^{{{{\rm{nr}}}}}\equiv {I}^{{{{\rm{nr}}}}}({S}^{(1)}\to {S}^{(2)})\cup {I}^{{{{\rm{nr}}}}}({S}^{(2)}\to {S}^{(3)})\cup \cdots \cup {I}^{{{{\rm{nr}}}}}({S}^{(q-1)}\to {S}^{(q)})$. Here *I*^nr^(*S*^(*ℓ*)^ → *S*^(*ℓ*+1)^) is the set of specific non-reciprocal interactions needed for the realization of the *S*^(*ℓ*)^ → *S*^(*ℓ*+1)^ transition, which is defined as

5 $$\begin{array}{l}{I}^{{{{\rm{nr}}}}}({S}^{(\ell )}\to {S}^{(\ell +1)})=\mathop{\bigcup}\limits_{\begin{array}{c}i,j\end{array}}\,\left\{\,{S}_{i-1,j}^{(\ell )}\swarrow {S}_{i,j}^{(\ell +1)},\right.\\ \left.{S}_{i,j}^{(\ell +1)}\nearrow {S}_{i+1,j}^{(\ell )},\,{S}_{i,j}^{(\ell +1)}\searrow {S}_{i,j-1}^{(\ell )},\,{S}_{i,j+1}^{(\ell )}\nwarrow {S}_{i,j}^{(\ell +1)}\,\right\}.\end{array}$$

The asymmetric interaction matrix *R*^nr^ contains those specific non-reciprocal interactions that are favoured by at least one of the transitions.

### Monte Carlo simulation

The introduction of non-reciprocal interactions into the multifarious self-assembly model renders the problem to have inherent non-equilibriumness. As such, a faithful treatment of the stochastic dynamics will require the use of an appropriate master equation formalism. To help highlight the connection with the equilibrium multifarious self-assembly model, however, we have chosen to use a generalized Monte Carlo scheme in which we have incorporated the non-reciprocal interactions in the spirit of kinetic Monte Carlo algorithms. Our specific implementation can be justified with the assumption of separation of timescales between the process of self-assembly and shape-shifting transitions.

In the lattice realization of our model, the whole system is defined as a square lattice of size $2\sqrt{M}\times 2\sqrt{M}$, in which the desired structures in the form of two-dimensional square lattices of size $\sqrt{M}\times \sqrt{M}$ would be embedded. We make use of the fully heterogeneous and zero-sparsity condition, that is, each component should appear only once in each structure^7^, and thus, each structure is a random permutation of the tiles in the square lattice. We follow a generalized version of the grand canonical Monte Carlo simulation as would have been implemented for the Hamiltonian ${{{\mathcal{H}}}}$, with the following generalization in the acceptance rate of every step. At each Monte Carlo step, a random lattice point (*i*, *j*) is chosen and its component *σ*_*i*,*j*_ is changed to another random component *σ*′ with probability

6 $$p=\min \left\{1,\exp \left({{\varLambda }}-{{\Delta }}{{{\mathcal{H}}}}\right)\right\},$$

where

7 $${{{\varLambda }}}_{i,j}={R}_{{\sigma }_{i-1,j}\swarrow {\sigma }^{\prime}}^{{{{\rm{nr}}}}}+{R}_{{\sigma }^{\prime}\nearrow {\sigma }_{i+1,j}}^{{{{\rm{nr}}}}}+{R}_{{\sigma }^{\prime}\searrow {\sigma }_{i,j-1}}^{{{{\rm{nr}}}}}+{R}_{{\sigma }_{i,j+1}\nwarrow {\sigma }^{\prime}}^{{{{\rm{nr}}}}}.$$

Evidently, in the limit *λ* → 0, this model reduces to the equilibrium multifarious self-assembly model as defined in other work^6,7^.

### Error calculation

The error of assembly is calculated as the fraction of extra tiles attached to the pattern or the fraction of tiles missing from the desired patterns. The error is defined as 1 − *O*, where *O* stands for overlap and is calculated as follows: (1) we find the largest connected cluster of the tiles *L*; (2) we then construct *A* = *L* ∪ *S*^(*i*)^, that is, the union of the desired structure as located at the centre of the lattice *S*^(*i*)^ and *L*; (3) we calculate *O*_*i*_ = ∣*A* ∩ *S*^(*i*)^∣/∣*A*∣. The error of self-assembly is *e*_*i*_ = 1 − *O*_*i*_, which is defined for a single desired structure. If a sequence is encoded in the system and if the system is initialized with one of the patterns in this sequence, then for sufficiently strong *λ*, we expect to observe shifts. Consequently, one needs to repeat step (3) for each of the patterns in the sequence and define error as ${{{\rm{error}}}}=\min \{{\overline{e}}_{i}\}{| }_{i}$, where *i* runs over all the patterns in that sequence.

### Colouring

We have used a convention for colouring the final configurations at the end of the simulations to be able to visually observe the self-assembled structures as well as transition dynamics. The colouring convention works as follows. First, we assign independent colours to each of the *m* desired structures. In the final configuration at the end of the simulation, a lattice point is either empty, which is then coloured white, or it is filled with a tile, which should then be coloured. A tile is coloured with respect to its nearest neighbours, and consequently, it can take only one of the *m* colours corresponding to the *m* desired structures. For example, let us consider a tile that has four neighbouring tiles specifically interacting with it. Since all the specific interactions are initially extracted from the stored structures, each of these four interactions can belong to one (or more) of the stored structures. For the tile under consideration, we select the colour of the structure that has the maximum contribution to its set of interactions with the neighbours. In the case of a tie, we randomly choose a colour from the colours of the competing structures. In the case when the tile makes specific bonds with none of its neighbours, then it should randomly inherit the colour from one of the structures. In some of the figures with many snapshots, to differentiate the liquid from chimera structures, we have coloured every tile if it specifically interacts with at least one of the neighbours.

### Brownian dynamics simulation

A more realistic model is introduced in the Supplementary Information. We define a box with a side length of ${N}_{x},{N}_{y},{N}_{z}=(2\times \sqrt{M},2\times \sqrt{M},2\times \sqrt{M}+1)$, which contains the pool of tiles that undergo stochastic motion. To make the comparison between this simulation and the Monte Carlo simulation, we implement the directional interactions on small cubic tiles that undergo translational Brownian motion in discrete space and discrete time. Tiles are locally interacting with excluded volume interactions in the entire box. They also interact with specific reciprocal and non-reciprocal interactions in the interaction region. The interaction region has the same size of a pattern placed in the middle of the box. At *t* = 0, we place an initial seed in the middle of the box. Tiles that reach the surface of the initial seed at *z* = *z*_seed_ ± 1 interact with the tile in the seed and replace it (provided the excluded volume condition is fulfilled) with a rate that follows the same probability as defined in the case of the kinetic Monte Carlo simulation, namely, $p=\min \{1,{\mathrm{e}}^{{{\varLambda }}-{{\Delta }}{{{\mathcal{H}}}}}\}$ . Note that the assumption of the existence of a region for the interactions is to avoid the nucleation of another structure that would lead to the depletion of tiles, which can consequently prevent shifting. We expect this assumption not to be restrictive, since for larger systems, the nucleation time in other areas will be longer than the time needed for a retrieval or shift in an existing seeded cluster and there will be enough tiles to nucleate many copies of the same structure at different places.

## Online content

Any methods, additional references, Nature Portfolio reporting summaries, source data, extended data, supplementary information, acknowledgements, peer review information; details of author contributions and competing interests; and statements of data and code availability are available at 10.1038/s41565-022-01258-2.

### Supplementary information

Supplementary Information Supplementary Figs. 1–20, Table 1, discussion and notes.

## Data Availability

The data supporting the main findings of this study are available in the Article and its Supplementary Information. Any additional data are available from the corresponding author upon request. Source data are provided with this paper.

## Source data

Source Data Fig. 2 Monte Carlo simulation output.
Source Data Fig. 3 Monte Carlo simulation output.
Source Data Fig. 4 Monte Carlo simulation output.

## Code availability

The algorithms for the codes supporting the main findings of this study are available in the Article and its Supplementary Information. Any additional information concerning the code is available from the corresponding author upon request.

## References

[1] Glotzer, S. C. Some assembly required. *Science* **306**, 419–420 (2004). 10.1126/science.1099988 15486279

[2] Hormoz, S. & Brenner, M. P. Design principles for self-assembly with short-range interactions. *Proc. Natl Acad. Sci. USA* **108**, 5193–5198 (2011). 10.1073/pnas.1014094108 PMC3069168 21383135

[3] Whitelam, S. & Jack, R. L. The statistical mechanics of dynamic pathways to self-assembly. *Annu. Rev. Phys. Chem.* **66**, 143–163 (2015). 10.1146/annurev-physchem-040214-121215 25493714

[4] Nguyen, M. & Vaikuntanathan, S. Design principles for nonequilibrium self-assembly. *Proc. Natl Acad. Sci. USA* **113**, 14231–14236 (2016). 10.1073/pnas.1609983113 PMC5167149 27911789

[5] Rao, A. B. et al. Leveraging hierarchical self-assembly pathways for realizing colloidal photonic crystals. *ACS Nano* **14**, 5348–5359 (2020). 10.1021/acsnano.9b07849 PMC7304928 32374160

[6] Murugan, A., Zeravcic, Z., Brenner, M. P. & Leibler, S. Multifarious assembly mixtures: systems allowing retrieval of diverse stored structures. *Proc. Natl Acad. Sci. USA* **112**, 54–59 (2015). 10.1073/pnas.1413941112 PMC4291664 25535383

[7] Sartori, P. & Leibler, S. Lessons from equilibrium statistical physics regarding the assembly of protein complexes. *Proc. Natl Acad. Sci. USA* **117**, 114–120 (2020). 10.1073/pnas.1911028117 PMC6955335 31871201

[8] Keim NC, Paulsen JD, Zeravcic Z, Sastry S, Nagel SR (2019). Memory formation in matter. Rev. Mod. Phys..

[9] Hopfield, J. J. Neural networks and physical systems with emergent collective computational abilities. *Proc. Natl Acad. Sci. USA* **79**, 2554–2558 (1982). 10.1073/pnas.79.8.2554 PMC346238 6953413

[10] Amit, D. J. *Modeling Brain Function: The World of Attractor Neural Networks* (Cambridge Univ. Press, 1992).

[11] Musacchio, A. & Salmon, E. D. The spindle-assembly checkpoint in space and time. *Nat. Rev. Mol. Cell Biol.* **8**, 379–393 (2007). 10.1038/nrm2163 17426725

[12] Golestanian, R. Phoretic active matter. Preprint at *arXiv* https://arxiv.org/abs/1909.03747 (2019).

[13] Soto R, Golestanian R (2014). Self-assembly of catalytically active colloidal molecules: tailoring activity through surface chemistry. Phys. Rev. Lett..

[14] Soto R, Golestanian R (2015). Self-assembly of active colloidal molecules with dynamic function. Phys. Rev. E.

[15] Saha S, Ramaswamy S, Golestanian R (2019). Pairing, waltzing and scattering of chemotactic active colloids. New J. Phys..

[16] Agudo-Canalejo J, Golestanian R (2019). Active phase separation in mixtures of chemically interacting particles. Phys. Rev. Lett..

[17] Ouazan-Reboul, V., Agudo-Canalejo, J. & Golestanian, R. Non-equilibrium phase separation in mixtures of catalytically active particles: size dispersity and screening effects. *Eur. Phys. J. E* **44**, 113 (2021). 10.1140/epje/s10189-021-00118-6 PMC8416889 34478002

[18] Uchida N, Golestanian R (2010). Synchronization and collective dynamics in a carpet of microfluidic rotors. Phys. Rev. Lett..

[19] Gong Z (2018). Topological phases of non-Hermitian systems. Phys. Rev. X.

[20] Saha S, Agudo-Canalejo J, Golestanian R (2020). Scalar active mixtures: the nonreciprocal Cahn-Hilliard model. Phys. Rev. X.

[21] You, Z., Baskaran, A. & Marchetti, M. C. Nonreciprocity as a generic route to traveling states. *Proc. Natl Acad. Sci. USA* **117**, 19767–19772 (2020). 10.1073/pnas.2010318117 PMC7444273 32753380

[22] Loos SAM, Klapp SHL (2020). Irreversibility, heat and information flows induced by non-reciprocal interactions. New J. Phys..

[23] Liu, Y. G. N., Jung, P. S., Parto, M., Christodoulides, D. N. & Khajavikhan, M. Gain-induced topological response via tailored long-range interactions. *Nat. Phys.* **17**, 704–709 (2021).

[24] Fruchart, M., Hanai, R., Littlewood, P. B. & Vitelli, V. Non-reciprocal phase transitions. *Nature* **592**, 363–369 (2021). 10.1038/s41586-021-03375-9 33854249

[25] Sompolinsky H, Kanter I (1986). Temporal association in asymmetric neural networks. Phys. Rev. Lett..

[26] Parisi G (1986). Asymmetric neural networks and the process of learning. J. Phys. A: Math. Gen..

[27] Denkov, N., Tcholakova, S., Lesov, I., Cholakova, D. & Smoukov, S. K. Self-shaping of oil droplets via the formation of intermediate rotator phases upon cooling. *Nature* **528**, 392–395 (2015). 10.1038/nature16189 26649824

[28] Zhang T, Wan D, Schwarz JM, Bowick MJ (2016). Shape-shifting droplet networks. Phys. Rev. Lett..

[29] Haas PA, Goldstein RE, Smoukov SK, Cholakova D, Denkov N (2017). Theory of shape-shifting droplets. Phys. Rev. Lett..

[30] Nagarkar A (2021). Elastic-instability–enabled locomotion. Proc. Natl Acad. Sci. USA.

[31] Seifert U (2012). Stochastic thermodynamics, fluctuation theorems and molecular machines. Rep. Prog. Phys..

[32] Bisker G, Polettini M, Gingrich TR, Horowitz JM (2017). Hierarchical bounds on entropy production inferred from partial information. J. Stat. Mech..

[33] Bisker, G. & England, J. L. Nonequilibrium associative retrieval of multiple stored self-assembly targets. *Proc. Natl Acad. Sci. USA* **115**, E10531–E10538 (2018). 10.1073/pnas.1805769115 PMC6233095 30348806

[34] Zeravcic Z, Manoharan VN, Brenner MP (2017). Colloquium: toward living matter with colloidal particles. Rev. Mod. Phys..

[35] Meng, G., Arkus, N., Brenner, M. P. & Manoharan, V. N. The free-energy landscape of clusters of attractive hard spheres. *Science* **327**, 560–563 (2010). 10.1126/science.1181263 20110500

[36] Derrida B, Gardner E, Zippelius A (1987). An exactly solvable asymmetric neural network model. EPL.

[37] Fukai T, Shiino M (1990). Asymmetric neural networks incorporating the Dale hypothesis and noise-driven chaos. Phys. Rev. Lett..

[38] Sahu, S., Yin, P. & Reif, J. H. A self-assembly model of time-dependent glue strength. in *Algorithmic Bioprocesses* (eds Condon, A., Harel, D., Kok, J. N., Salomaa, A. & Winfree E.) 185–204 (Springer, 2009).

[39] Zeravcic, Z. & Brenner, M. P. Spontaneous emergence of catalytic cycles with colloidal spheres. *Proc. Natl Acad. Sci. USA* **114**, 4342–4347 (2017). 10.1073/pnas.1611959114 PMC5410808 28396424

[40] Zhong, W., Schwab, D. J. & Murugan, A. Associative pattern recognition through macro-molecular self-assembly. *J. Stat. Phys*. **167**, 806–826 (2017).

