# Supplementary material for: Non-reciprocal multifarious self-organization
Source: Nat Nanotechnol. 2022 Dec 12;18(1):79–85. doi: 10.1038/s41565-022-01258-2 (PMC9879770; doi:10.1038/s41565-022-01258-2)
Supplement: Supplementary file 1 — Supplementary Figs. 1–20, Table 1, discussion and notes. [file 41565_2022_1258_MOESM1_ESM.pdf]

nature nanotechnology

Article https://doi.org/10.1038/s41565-022-01258-2

Non-reciprocal multifarious self-organization

In the format provided by the authors and unedited

Contents

1 Non-Reciprocal Multifarious Model **2**

1.1 Stored structures and sequences . . . 2
1.2 Calculating overlap and error . . . 3
1.3 Convex hull of the shape shifting regime . . . 4
1.4 Shifting frequency . . . 8
1.5 Capacity versus non-reciprocal coupling . . . 8
1.6 Seed instability . . . 11
1.7 Loose bounds on λ . . . 12
1.8 Time scales . . . 15
1.9 Entropy production . . . 17
1.10 Cycles . . . 21
1.11 Period of cycles . . . 23
1.12 Basins of attraction for cycles . . . 23

2 Brownian Dynamics Simulation **26**

1 Non-Reciprocal Multifarious Model

In our model small building blocks (tiles) are interacting specifically. Predefined target structures are encoded in the specific interactions. We showed that adding non-reciprocal flavour on top of these specific reciprocal interactions provide space to design and implement shape shifting structure, in that, predefined order of target structures can be realized. Here, we present additional details of the model and supplementary figures that support the results presented in the main text.

1.1 Stored structures and sequences

We consider a pool of M distinct tiles labeled from 1 to M. Desired structures are two dimensional square lattices of size $\sqrt{M} \times \sqrt{M}$ composed of M tiles. Each structure is a random permutation and arrangement of the tiles in the lattice, moreover, each tile appears only once in each structure. There are total number of m desired structures.

The goal is to implement shape-shifting structure and hence, one needs to define desired shifting sequences. Consider a single shape-shifting sequence of length $q-1$ which is an ordered sequence of q structures in a specific order $\{S^{(1)} \to S^{(2)} \to S^{(3)} \to \cdots \to S^{(q-1)} \to S^{(q)}\}$. The following rules apply to the sequences:

1. Every structure in the sequence belongs to the set of m predefined desired structures.
2. The sequence is made of unique structures (unless the sequence is closed as a loop which in that case the first and last structure in the queue are the same) which means each desired structure at most appears once in any sequence.
3. The Order of the structures in the sequence is important and different orders of the same structures give different sequences.
4. Whenever there is more than one sequence rule (2) still applies, in that, each desired structure can take part at most in one of the sequences and only once.

The system that accommodates the realization of self-assembly of structure or shifting of the sequence is a 2D square lattice of size $\sqrt{N} \times \sqrt{N}$, where $\sqrt{N} = 2\sqrt{M}$.

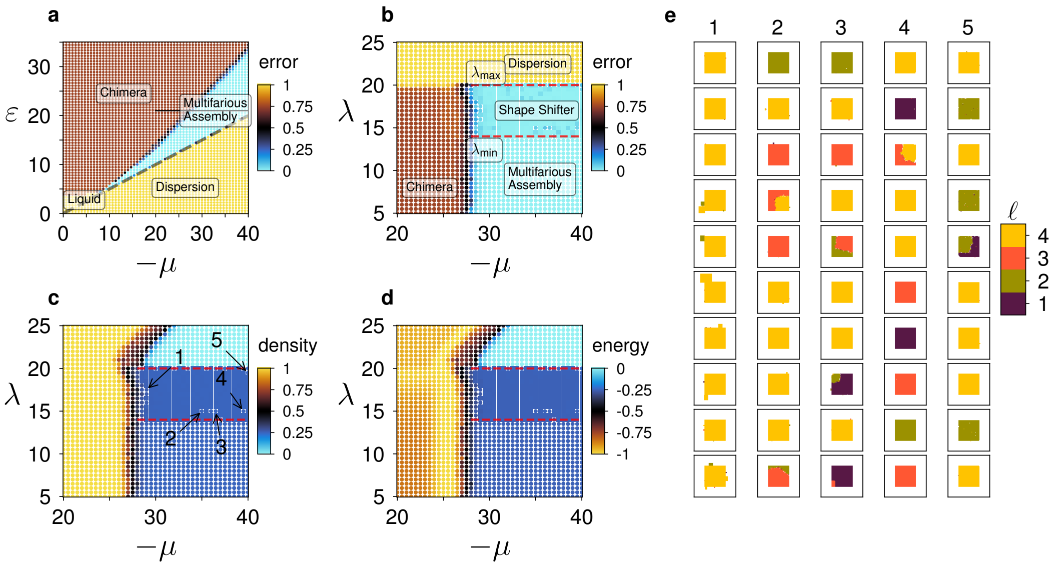

Figure S1: **Overestimation of error.** Panels **a-d** are the same as found in Fig. 3 of the main text, but complemented by the density and energy plots. In **c**, five candidate coordinates have been chosen. These points lie in the shape-shifting regime, although they are not classified correctly since they had higher error values. In **e**, all the snapshots of these five points are demonstrated. It is easy to see that point (1) belongs to the chimera regime. The other 4 points are in the shape-shifting regime, although a high error value is estimated due to the occurrence of the shift at the final steps of the simulation.

1.2 Calculating overlap and error

The final configuration at the end of the simulation should be compared with the ideal target structures in order to calculate the success of self-assembly. At the end of simulation, possible different outcomes are: complete retrieval of the target structure, partial retrieval in which either part of the target structure is not assembled or the structure is assembled with mistakes (chimeras), and other cases in which the structure is not stable and falls into small non-extensive pieces ending in dispersion or liquid regimes as discussed in the main text. To capture all these different outcomes, the three descriptors of error, density, and energy are used as "order parameters". Error is defined as $1 - O$, where O stands for overlap and is calculated as follows: (a) find the largest connected cluster of the tiles that we call L, (b) find $A = L \cup S^{(i)}$, i.e., the union of the desired structure located at the centre of the lattice $S^{(i)}$ and L, and (c) calculate $O_i = |A \cap S^{(i)}|/|A|$. Then, the error of self-assembly is $e_i = 1 - O_i$.

Calculating error in the case of shifting of a sequence needs some attention. Consider the sequence $\{S^{(1)} \to S^{(2)} \to S^{(3)} \to \cdots \to S^{(q-1)} \to S^{(q)}\}$. Seeding the simulation with $S^{(1)}$ and depending on the simulation parameters $(m, \lambda, \varepsilon, \mu, \mathcal{T}, q)$, we may or may not observe shifts. For example, in the parameter space of multifarious assembly regime, for a short simulation time $\mathcal{T}$, the time may not be sufficient to realize a shift and the system will then have a large overlap with the initial seed $S^{(1)}$. For sufficiently long time $\mathcal{T}$, the system may go through a different number of shifts. Hence, any of the structures in the sequence are considered a valid structure when evaluating error. In this regards, when calculating the error, 1) the final structure should be compared with each of the structures in the queue "starting from the seed structure", 2) the corresponding error values should be calculated, and

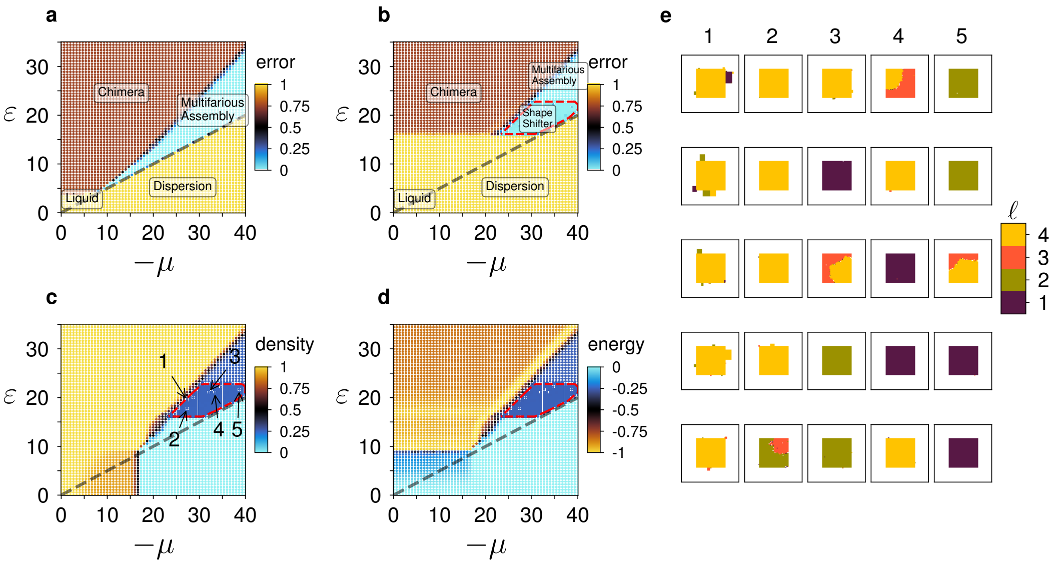

Figure S2: **Overestimation of error.** Panels **a-d** are the same as found in Fig. 3 of the main text, corresponding to (μ, ε) parameter scan, but with $\lambda = 15$. In **c**, five candidate coordinates have been chosen. These points lie in the shape-shifting regime, although they are not classified correctly since they had higher error values. In **e**, all the snapshots of these five points are demonstrated. It is easy to see that point (1) belongs to the chimera regime. The other 4 points are in the shape-shifting regime, although a high error value is estimated due to the occurrence of the shift at the final steps of the simulation.

3) the minimum value of these errors is chosen as the shifting error. In other words, a vector of errors is created where $\vec{e} = (e_1, e_2, e_3, \ldots, e_{q-1}, e_q)$ and, finally, error $= \min\{\bar{e}_i\}|_i$. In practice, one needs to calculate the error of the final configuration with respect to the structures in the sequence that lie beyond the initial seed in the predefined sequence. A potential issue with this definition can arise when $\mathcal{T}$ coincides with one of the shifting events. In this case, we may record a chimera structure composed of two consecutive structures in the sequence as shown in columns 2-5 in Fig. S1 and Fig. S2. While the algorithm reports a high value of error, indeed the error is still zero if one waits for the completion of the shift.

Error by itself may not be sufficient to determine the regime that the final configuration belongs to. For example, instances from dispersion, liquid and chimera regimes may have the same error values error ≈ 1. In these cases, one needs to monitor density and energy to differentiate the different outcomes. For example, an instance of dispersion regime comes with low density and high energy values whereas an instance of liquid regime comes with high density and high energy values. On the other hand, a chimera structure is always observed with high error, high density, and low energy values.

1.3 Convex hull of the shape shifting regime

In Fig. 3 of the main text, as well as Figs. S1-S2 here, the points in the parameter space of non-reciprocal multifarious self-organization model capable of realizing a shape shifting structure are identified with red

dashed lines. For each point in the parameter space, e.g. (μ, ε) or (μ, λ) the error is calculated using the method explained in the previous section and is averaged over independent realizations of the system. As argued in the main text, a shape shifting structure can only be realized in the sub-space of parameter space with multifarious assembly feature identified with nearly zero self-assembly error, namely, the cyan region in Fig. S1**a**. For sufficiently strong non-reciprocal coupling, a part of the multifarious assembly region converts to being a shape-shifting region.

To locate the shape shifting region within the multifarious assembly regime, the following procedure is used. First, within the multifarious assembly regime we consider the points with error < 0.05. Then, for these points the final configuration is considered for calculating the overlap with all the structures in the sequence $\vec{O} = (O_1, O_2, O_3, \ldots, O_{q-1}, O_q)$, and we define $O = \max\{O_i\}|_i$. If the maximum overlap corresponds to a structure other than the initial seed, it means that the system has undergone at least one shift. These points (with low error value for at least one shift) are identified with square markers in the figures, as e.g. in Fig. S1. The region covered by the different markers delineate the sub-space of parameter space with shape-shifting property; see Fig. S1**b**. To delineate all the regimes one has to take into account the density and energy as "order parameters". As an example, consider two representative points in liquid and dispersion regimes. The error value is the same for these two points; error ≈ 1. However, one is identified with high density (liquid) and the other with low density (dispersion). The same method can be used to differentiate liquid from chimera, in cases with error ≈ 1.

It is worth mentioning that one may see a few points in the multifarious regime that lie in the shape shifting regime while not picking up the different markers as shown in Fig. S1**c**. This can happen for two main reasons. First, close to the chimera regime (showed by arrow 1) a point can behave like shape shifter and chimera, as illustrated by some realizations in the first column in Fig. S1**e**. Second, this can arise due to the strict restriction that we applied by considering only the points with error < 0.05. The second group of points (arrow2-arrow5) indeed belong to the shape-shifting regime. However, the time of measurement by chance has coincided with the transition time as shown in some of the realizations in Fig. S1**e**, and thus a high value of error is estimated for these points (as discussed above).

We used the convex hull to capture borders of shape shifting regime. Consider a parameter space of the model, e.g. (μ, ε) space in Fig. S2**a**. In the main text, we have shown that a sufficiently strong non-reciprocal coupling can partially convert multifarious assembly regime to a shape-shifting regime. Figures S2**b**, **c**, and **d** show this for $\lambda = 15$. The red dashed line which functions as the limiting border of the shape shifting regime is obtained as follows. First, all the points in multifarious assembly regime with the strict limit of error < 0.05 are selected. Now, within this set we only keep the points that have undergone at least one shift in one of the realizations as discussed before. Then, the convex hull of the new set is labeled as the shape-shifting regime by red dashed line. As mentioned above, the coincidence of measurement time and shifting time can occur also in this case, as shown in Fig. S2**c** with black arrows and the corresponding snapshots in panel (e).

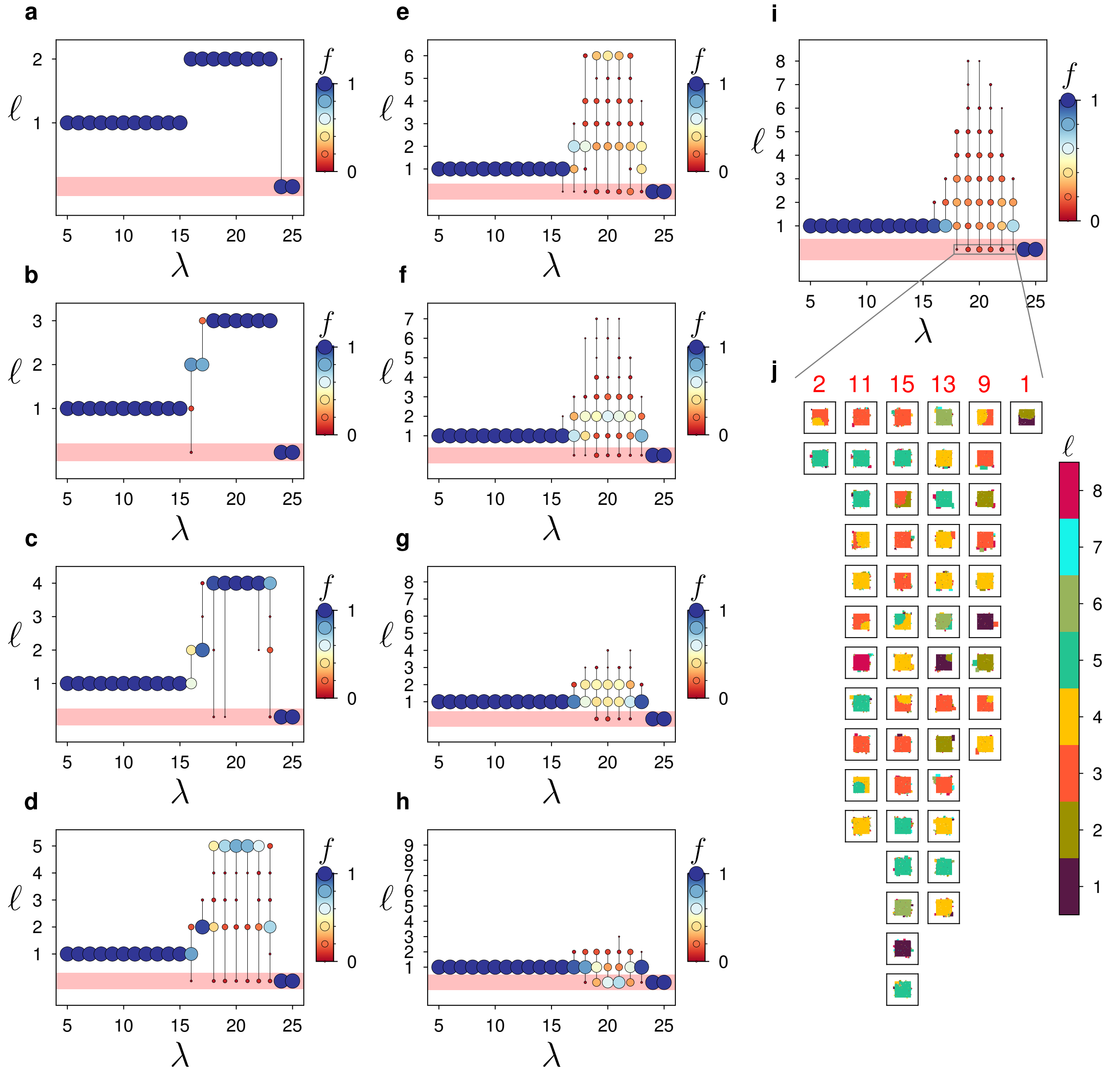

Figure S3: **Dependence of the shifting frequency on the length of the sequence.** Panels **a-h** show the shifting frequency for different lengths of the stored sequence. While the window defined by λ_{min} and λ_{max} is almost fixed for different lengths, the pattern of shift changes. Increasing the length of the sequence beyond the storage memory can lead to erroneous structures, as illustrated in **h**. As we increase m from **a** to **h**, intermediate patterns are more populated with different realizations of the system. Storing longer sequences needs more time to allow the system to reach the patterns located at the end of the sequence. This can be seen from **i**, which is the same as **g** but simulated for much longer times. Panel **j** shows all the structures recorded as erroneous in **i**. Most cases correspond to the overlap of measuring time and transition time.

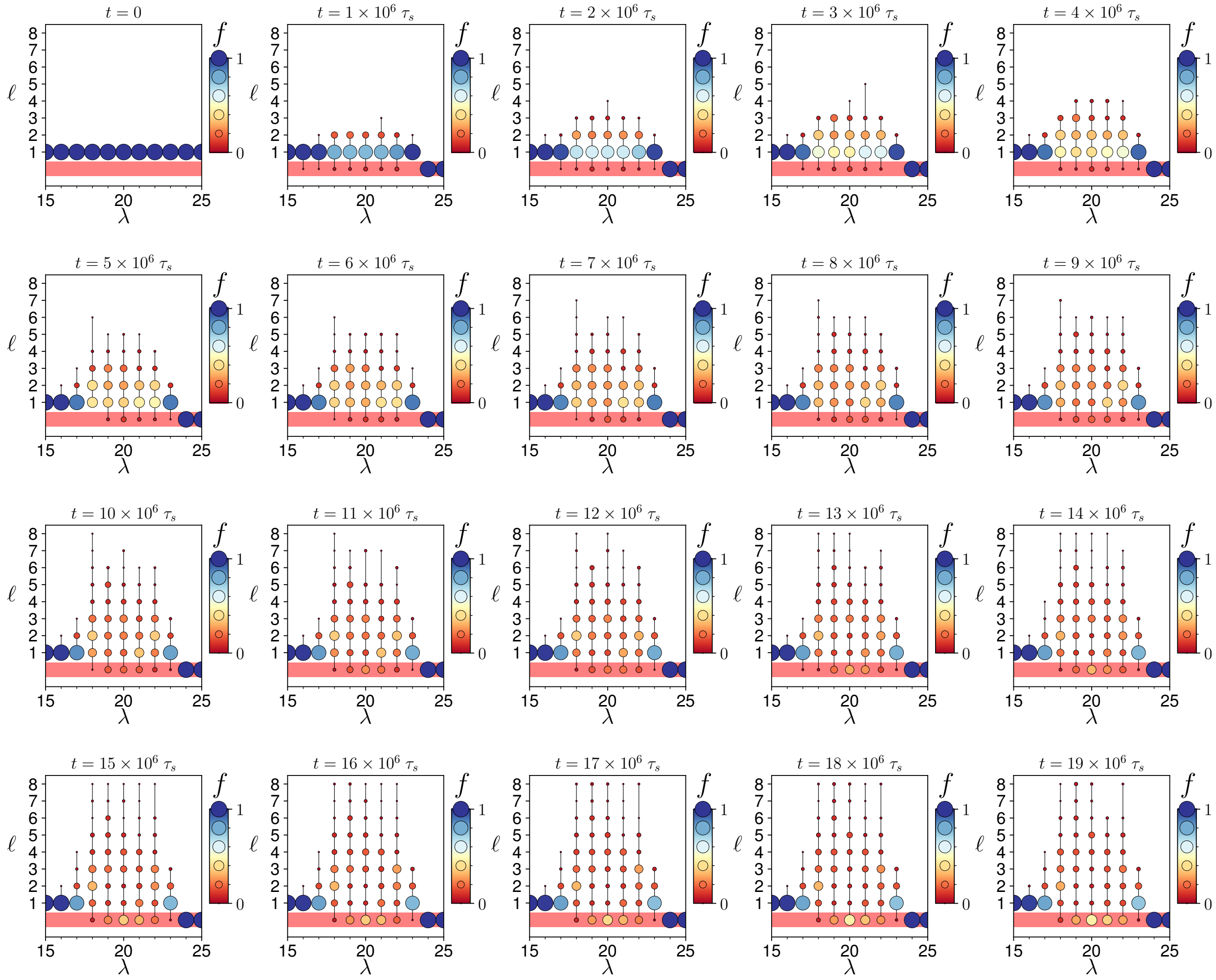

Figure S4: **Time evolution of the shifting frequency.** A sequence of length 7 composed of 8 structures is stored in the system with (μ, ε) coordinates deep in the multifarious assembly regime [$\diamond$ marker in Table 1]. The frequencies are recorded in time steps $10^6\tau_\mathrm{s}$. Initializing the realizations from the first structure, sufficient non-reciprocal drive and time enable the system to switch to the structures in the sequence up to the last structure.

marker	ε	μ	λ
$\square$	10.55555556	18.0	8.5
$\star$	12.22222222	20.66666667	10.0
$\triangledown$	13.88888889	23.33333333	11.0
$\triangle$	15.55555556	26.0	12.0
$\triangleleft$	17.22222222	28.0	13.0
$\triangleright$	18.88888889	30.66666667	14.0
$\bigcirc$	20.55555556	33.33333333	17.0
$\Diamond$	22.22222222	36.0	17.0
$\diamond$	24.44444444	38.66666667	19.0

Table 1: Coordinates of the points in multifarious assembly regime used in different figures and λ used for their shape-shifting realizations.

1.4 Shifting frequency

To demonstrate the frequency of the shifts we use the multi-bulleted bar as in Fig. S3. Let us consider implementing the following sequence $\{S^{(1)} \to S^{(2)} \to S^{(3)} \to \cdots \to S^{(q)}\}$. For a specific value of λ, we perform 100 independent simulations of the system seeded with initial structure $S^{(1)}$. Then, the frequency vector $\vec{f} = (f_0, f_1, f_2, \ldots, f_q)$ is built where f_ℓ is the fraction of realizations that (at the time of recording) had the minimum error (or maximum overlap) with respect to structure ℓ. If the minimum value of error is ≥ 0.1 it is classified as an erroneous structure and the corresponding frequency is represented by f_0. Now, for each value of λ there is a $\vec{f}$, which we display as multi-bulleted bars, with the bullets appearing at the structures with non-zero frequency $f \neq 0$. Figure S3 shows the frequency plot for different stored structure for the same simulation time $10^6\tau_s$. As the length of the sequence is increased, more and more intermediate structures appear in the bulleted bars. In this regards, it is illuminating to compare panels **g** and **i** in the same figure. **i** is the same as **g** realizing a sequence of length 7 (8 structures) but for a longer time $\mathcal{T} = 10^7\tau_s$, which shows that for longer sequences longer simulation times are needed for capturing the structures at the end of the sequence. The red bands and the bullets that fall in the red band correspond to f_0 or erroneous structures. Panel **j** shows all the erroneous configurations corresponding to the red band in **i**. The snapshot in **j** shows that many of the configurations indeed are not erroneous, and that the coincidence of the simulation time $\mathcal{T}$ and the shifting time can lead to erroneous classification.

We now turn to the time evolution of the frequencies, as shown in Fig. S4. Frequencies are shown for a length 7 sequence (8 structures) stored in the system with (ε-μ) deep in the multifarious assembly regime [corresponding to $\diamond$ in Table 1]. The frequencies are recorded with the time step of $10^6\tau_s$. As time goes on, more and more of the higher structures in the sequence are occupied in the realizations of the system. Note that we have started in **a** with all the realizations initialized by the first structure.

1.5 Capacity versus non-reciprocal coupling

It would be interesting to explore what happens when we change the number of stored structures m. We have already shown that all the multifarious assembly region potentially provides a design space for shape shifting behaviour for appropriate choice of λ. Let us consider a choice of (μ, ε) deep in the multifarious assembly regime corresponding to the diamond marker in Fig. 4**a**. For this fixed value of energy and

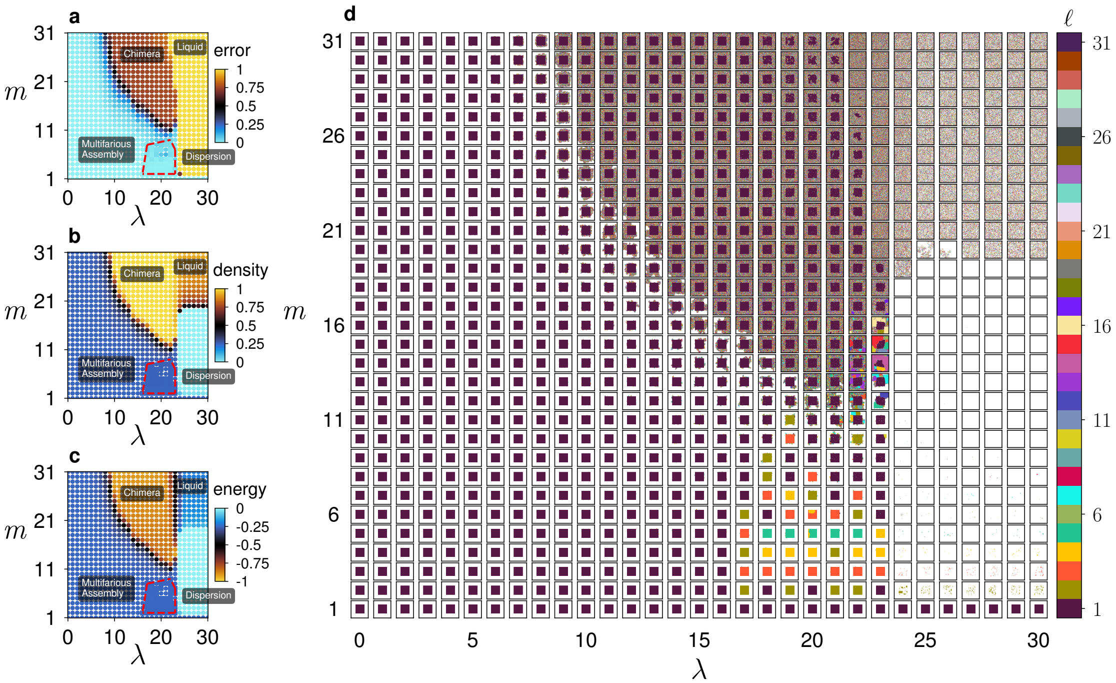

Figure S5: **Capacity versus non-reciprocal coupling.** The behaviour of the system is probed for different values of desired structure number m and sequence length $m-1$ (with structures of size 40×40). For each set of (m,λ) we have introduced the complete initial structure as seed and simulated the system with 10 independent realizations of the system for $4\times10^6\tau_s$ steps. Panels **a**, **b** and **c** show the average error, density, and energy, respectively. Sufficiently strong non-reciprocal interactions introduce the shape shifting behaviour. The red dashed line is the convex hull of the points that correspond to error < 0.05 and have undergone at least one shift. **d**, The last snapshots of the simulation for each (m,λ) pair.

chemical potential, we have mapped out the (m,λ) parameter space, as shown in Fig. S5 and S6). Sequences of different lengths from $m=1$ (a single structure) to $m=31$ (31 structures connected as a sequence of length 30) are stored in the system. The system is then realized independently for different values of λ, from $\lambda=0$ to $\lambda=30$ from the initial structure, $\ell=1$. For each parameter pair (m,λ) 10 independent realizations of the system is implemented and the three descriptors of error, density and energy, as shown in Figs. S5**a**, **b**, and **c**, respectively, are used for determining the behaviour of the system. Figure S5**d** shows the last of the simulation, which further clarifies the differences between the phases. As we can anticipate, weak non-equilibrium drive does not affect the capacity of the system, as it can successfully restore (or keep) the initial seed, and consequently, shape-shifting is not observed. Moreover, we observe the shape-shifting behaviour within the band $(\lambda_{\text{min}},\lambda_{\text{max}})$, and delineated by the red dashed line as convex hull of the points that have undergone at least one shift (see Fig. S6 for more details).

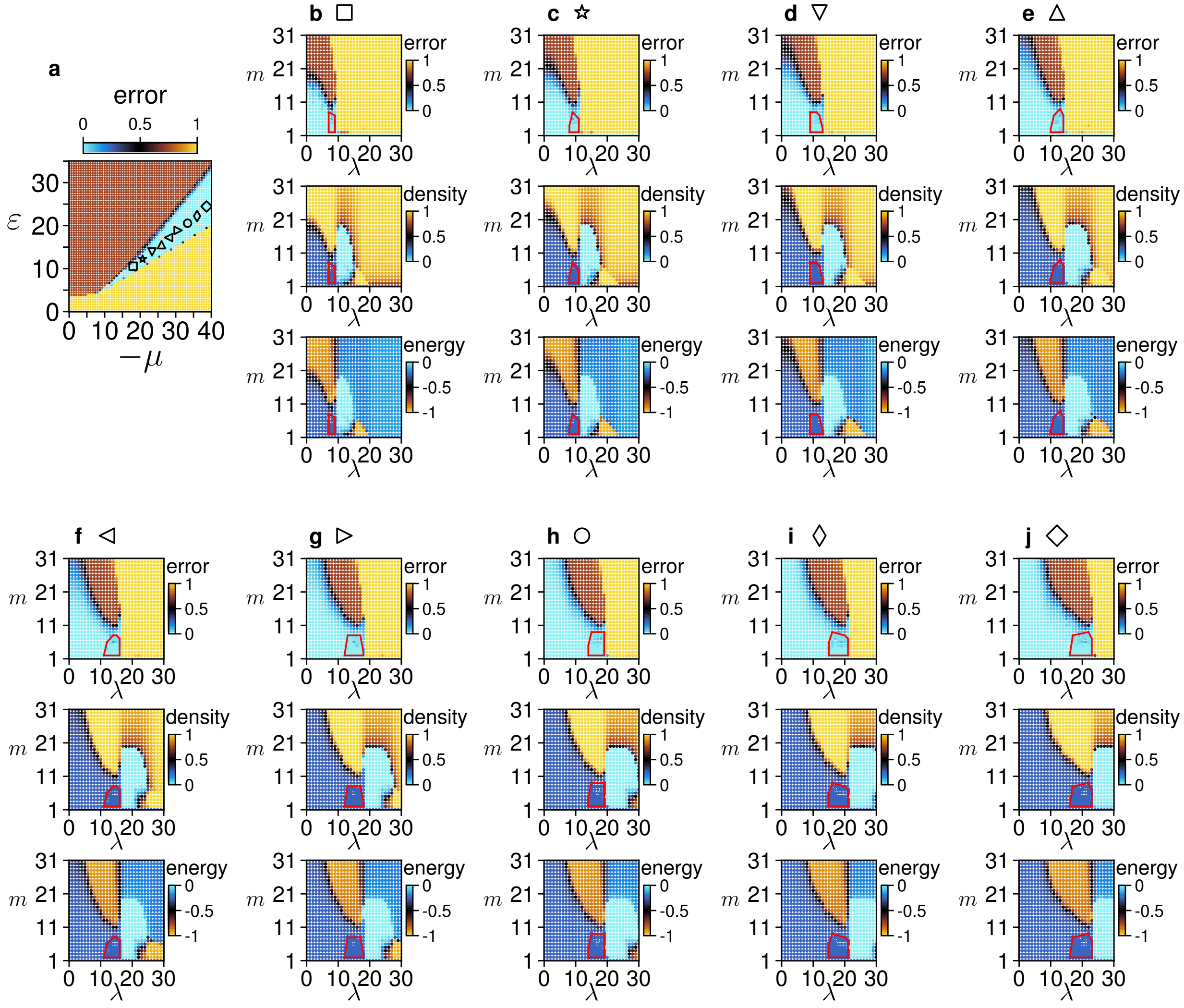

Figure S6: **Available parameter space to implement shape shifters.** **a**, Selection of coordinates in (μ, ε) space that starts from the edge of the multifarious assembly regime close to the dispersion-liquid-chimera intersection (square marker) and moves in towards the multifarious assembly regime (Diamond marker). Each 3-rowed panel, **b**-**j**, shows (m, λ) parameter scan of the non-reciprocal multifarious self-organization model corresponding to the (μ, ε) coordinates in **a**, encoding sequences of length $m-1$. One observes that the volume of the parameter space affording multifarious assembly regime, and consequently, that of the shape-shifting regime, increase as one moves deeper in the multifarious assembly regime; **b**-**j**. The red line delineating the shape-shifting regime is the approximate convex hull of all the points that are subsets of the multifarious assembly regime, and have undergone at least one shift. It is clear that the shape-shifting property is obtained at the cost of capacity reduction.

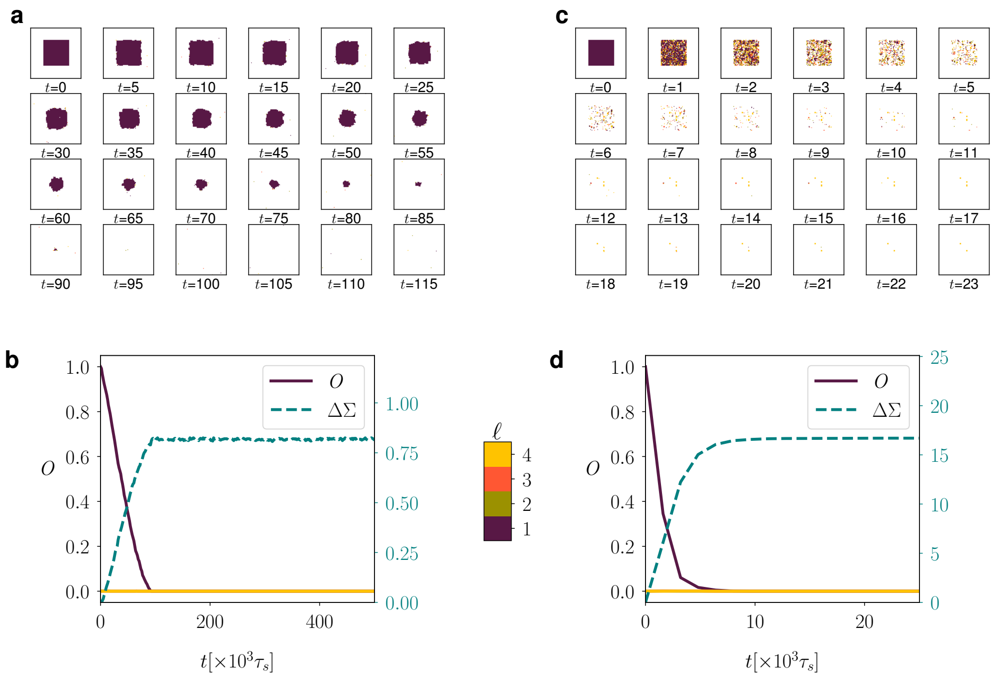

Figure S7: **Seed instability.** Seed instability at equilibrium (i.e., only reciprocal bonds are present) corresponding to $-\mu > 2\varepsilon$ as well as the non-reciprocal case, corresponding to $\lambda > \varepsilon$. 4 structures are stored in the system with $(\mu, \varepsilon, \lambda) = (-15, 6, 0)$ for the equilibrium case (**a** and **b**) and a sequence of length 3 composed of 4 structures is stored with $(\mu, \varepsilon, \lambda) = (-20, 11.5, 12)$ for the non-reciprocal case (**c** and **d**). **a**, Snapshots of the system at different times during the dispersion process under equilibrium conditions. **b**, Overlap and entropy production associated with the system in **a**. Panels **c** and **d** are the same as **a** and **b** but for the non-reciprocal case.

1.6 Seed instability

In the main text we referred to a range of $\lambda \in [\lambda_{\min}, \lambda_{\max}]$ where a shape-shifter is realizable. We mentioned that increasing λ beyond the value of $\lambda_{\max}$ results in the instability of the original seed. Here we present an instance of this instability and compare it to the instability or melting in the equilibrium case. Let us consider a case where the system is initialized at equilibrium with one of the stored pattern as seed. We consider a tile at the corner of the structure and note that it makes two specific bonds with its immediate neighbours. At equilibrium, a tile can justify sitting at a grid point if it compensates its presence with correct specific interactions with the neighbours. In this case, a corner tile becomes unstable if $2\varepsilon < -\mu$. This can initiate melting of the structure from outer layers as shown in Figs. S7**a** and **b**. We now focus on a case where seed is stable from the point of view of equilibrium condition, i.e., $2\varepsilon \geq -\mu$, but in the presence of a strong non-reciprocal drive $\lambda > \varepsilon$. This condition makes each lattice point a potential seed to start the shift. However, the time of shifts are so fast that the system cannot wait to pick up the right tiles. Therefore, it grows to induce a shift, culminating in the occurrences of shifts within shifts until it becomes unstable, as shown in Figs S7**c** and **d** .

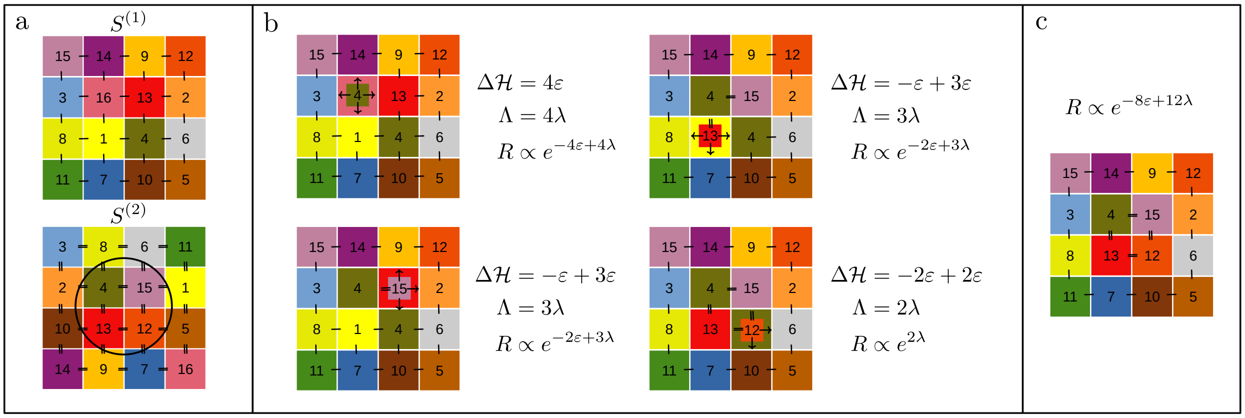

Figure S8: **Loose bounds on λ_{min} and λ_{max}.** Panel **a** shows two desired patterns encoded as a sequence of length one; $S^{(1)} \to S^{(2)}$ in the interaction matrices. **b**, Starting of shift by nucleating a small seed of $S^{(2)}$ within $S^{(1)}$. Each step shows the energetic factor with the added non-reciprocal contribution. Panel **c** shows the final weight for formation of the small square seed.

1.7 Loose bounds on λ

We observe that for a given (μ, ε) a shape-shifting state can appear for some values of $\lambda \in [\lambda_{\mathrm{min}}, \lambda_{\mathrm{max}}]$. Here, we present a simple argument to get loose bounds on the strength of non-reciprocal interactions λ_{min} and λ_{max}. Let us consider the case shown in Fig. S8 where two patterns $S^{(1)}$ and $S^{(2)}$ and the corresponding shifts $S^{(1)} \to S^{(2)}$ are programmed in the interaction matrices. While there are different kinds of realizations of shifts, here we consider a case where a shift realization starts with nucleation of a small square seed of $S^{(2)}$ within $S^{(1)}$, as triggered by the non-reciprocal interactions. Figure S8**b** shows four required transitions with their corresponding rates. Ignoring entropic factor, we can set the exponential weight to ≈ 1 and estimate a loose bound of $\lambda_{\mathrm{min}} = \frac{2}{3}\varepsilon$ to initiate a shift. This mechanism can break down if the drive is strong enough to prevent stable formation of seed. A possible scenario can be realized when each lattice point by itself can initiate the shift. This can happen when the weight of the first step in Fig. S8**b** is ≈ 1, which gives us the next loose bound $\lambda_{\mathrm{max}} = \varepsilon$. Note that the presence of a lower bound on λ does not mean that shifts will be absent for $\lambda < \lambda_{\mathrm{min}}$. This bound is valid due to a limited observation time. Sufficiently long observation times will increase the probability of shifts for weak λ. Figure S9 shows that these bounds capture the limiting values of λ relatively well, for different pattern sizes. This is consistent with the overall observation that the behaviour of the model is robustly reproduced when the system size changes, as can be observed in Fig. S10 for 70×70 patterns, provided we make adjustments to the relevant time scales. The next section addresses this notion in details.

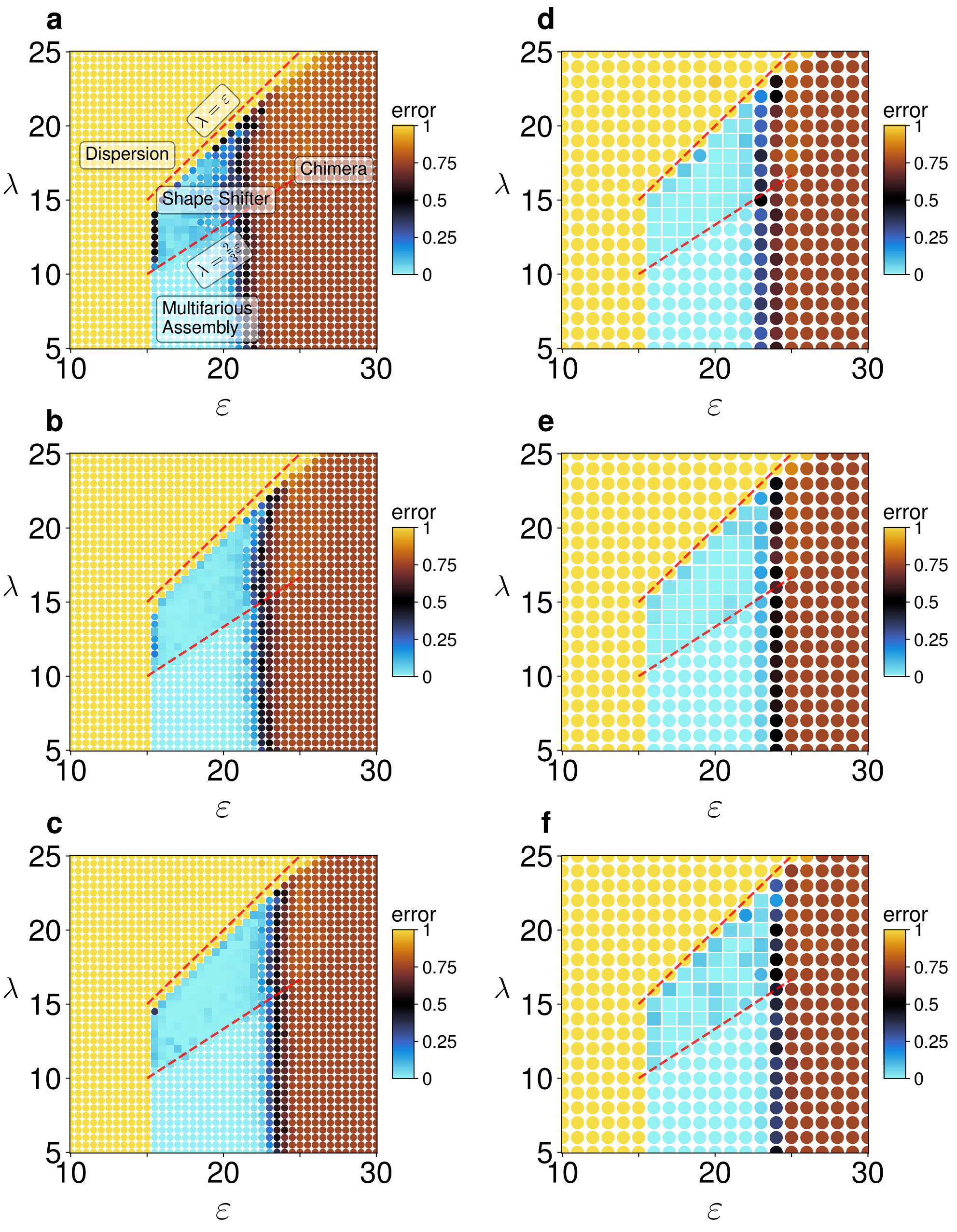

Figure S9: **Scaling with system size.** For a fixed $\mu = -30$ we explore the (λ, ε) parameter space. Four patterns are encoded as a sequence of length three where $S^{(1)} \to S^{(2)} \to S^{(3)} \to S^{(4)}$. Panels **a** to **f** correspond to patterns with sizes 20×20, 30×30, 40×40, 50×50, 60×60, and 70×70, respectively. The corresponding system sizes can be obtained by multiplying each pattern side length by a factor of two. Simulation times are $t = 4 \times 10^6 \tau_s$ and $t = 8 \times 10^6 \tau_s$ for left and right columns, respectively. Each marker is obtained by averaging over 10 independent realizations. All points with error ≤ 0.1 are considered for shifting property, i.e., if a point has ended in a configuration other than the initial seed it is considered a shift and a square marker replaces the corresponding circle marker. Red dashed lines are the loose bounds introduced in Fig. S8.

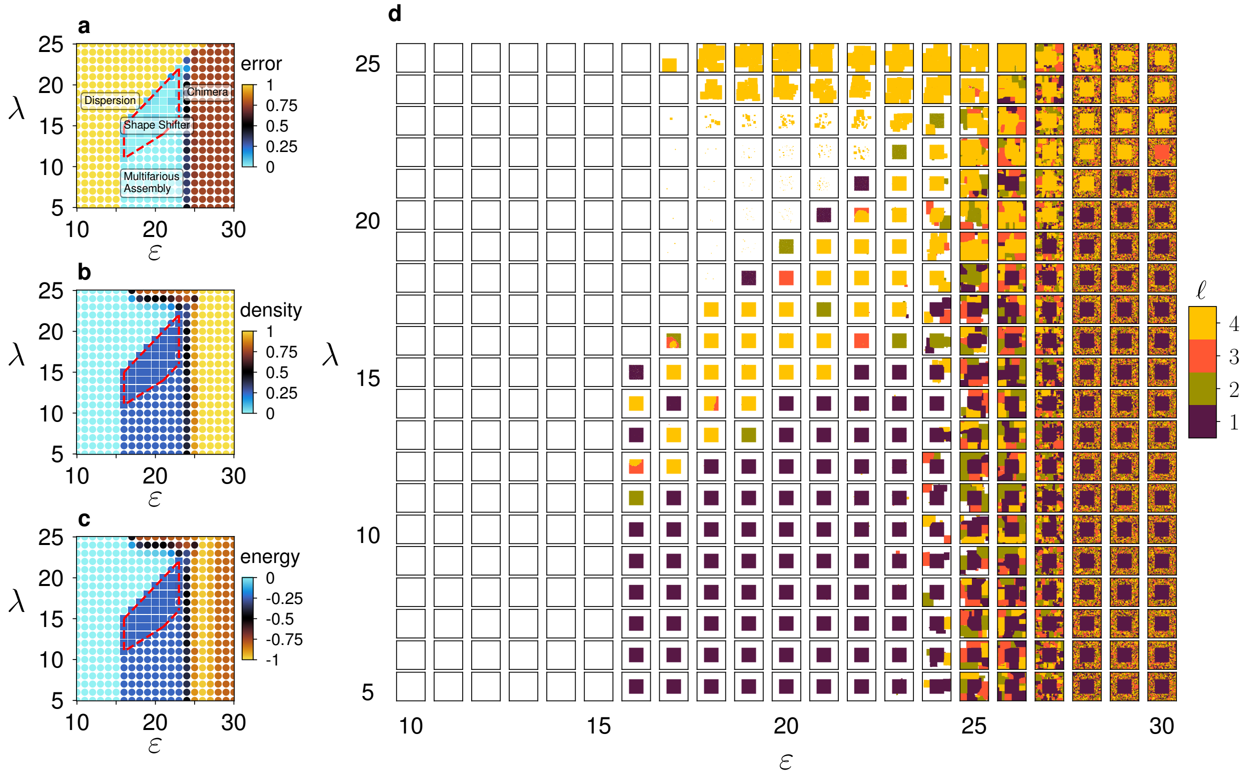

Figure S10: **Emergence of shape-shifters in** 70×70 **patterns.** For fixed $\mu = -30$ we explore (λ, ε) parameter space. Four patterns of size 70×70 are encoded in a sequence of length 3 $S^{(1)} \rightarrow S^{(2)} \rightarrow S^{(3)} \rightarrow S^{(4)}$. Panels **a**, **b**, **c** show error, density, and energy, respectively, after $8 \times 10^8 \tau_s$ simulation steps. Panel **d** shows a single snapshot for each set of $(\lambda,\, \varepsilon)$. Notice the colourful patterns retrieved at regions corresponding to the convex hull reported in **a**.

1.8 Time scales

The difference in the mechanism of shifts, which employ non-reciprocal interactions, and the equilibrium retrieval mechanism, which is based on reciprocal interactions can be probed through the relevant time scales.

In the equilibrium multifarious-assembly model, we define *r*etrieval as the process of complete recovery of a pattern starting from an initial seed of size 25% of the total pattern. Such a large seed is chosen in order to explore the time scale up to pattern sizes as large as 100×100. We can thus define the time scale of retrieval $\tau_{\text{retrieval}}$ as the time needed for the overlap to increase from 0.25 to values higher than 0.95. We set a small margin to avoid overestimation of the time due to attachment of a small number of incorrect tiles or missing a few correct tiles in otherwise almost completely retrieved cases.

In non-reciprocal multifarious model we refer to *s*hift as the process of starting a transition from one pattern (i.e., decreasing overlap from 0.95 to 0) to the other pattern (i.e., increasing overlap from 0.0 to 0.95) and the relevant time scale is also defined as τ_{shift}. See the first two panels in Fig. S11 **a** for the definition of $\tau_{\text{retrieval}}$ and τ_{shift}. In contrast to Fig. 4 of the main text, here we used the same small seeds in the presence of non-reciprocal interactions ($\lambda = 8$). Here, retrieval and shift compete. As expected, the initial seed may have the chance to retrieve the structure before the shifts are initiated. Figure S11**a** shows this case for patterns of size 100×100. Figure S11**b** shows the case where retrieval and shifts are happening together, leading to the correct final configuration.

Figure S11 shows the scaling of $\tau_{\text{retrieval}}$ and τ_{shift} with the size of the pattern. A clear distinction between the mechanisms of retrieval and shift can be observed in their corresponding time scales. $\tau_{\text{retrieval}}$ scales approximately as a power-law with the dynamic exponent $z = 2$ as expected. Note that to calculate $\tau_{\text{retrieval}}$ only the fully retrieved cases like the ones in Fig. S11**a** have been used.

Finally, we examine the fraction of instances where completely retrieved structures undergo shift. Figure S11**d** shows the number of these cases divided by the total number of realizations. Even for this moderate value of λ and large patterns such as 100×100, a considerable fraction of realizations can achieve complete retrieval before undergoing any shifts.

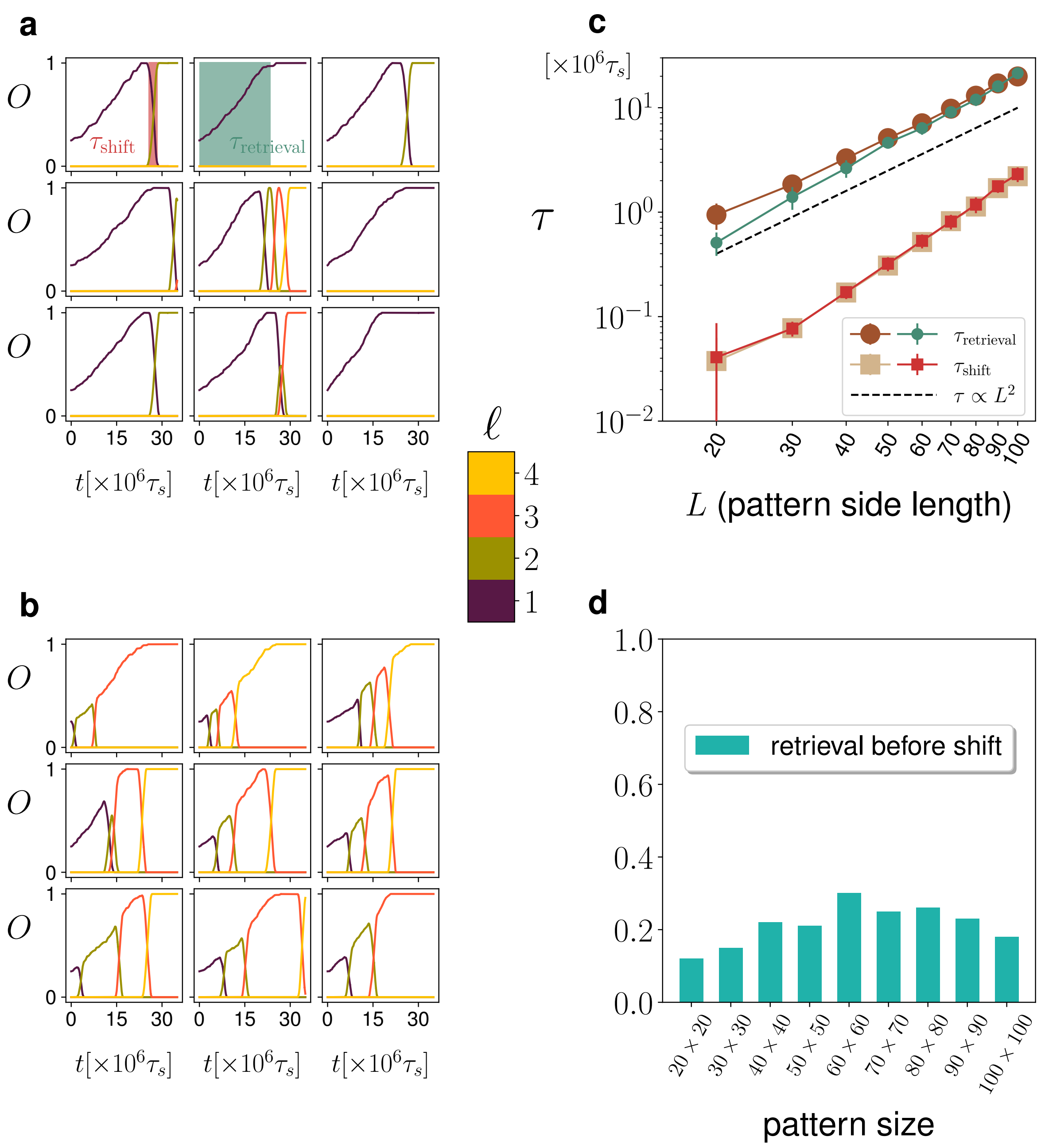

Figure S11: **Shape-shifting vs multifarious-assembly time scales.** **a**, 9 different realizations of complete retrieval of patterns of size 100×100 from a seed of size 50×50 in the presence of sufficiently strong non-reciprocal coupling. **b**, 9 different realizations of retrieval when accompanied by shifts. **c**, Scaling of $\tau_{\text{retrieval}}$ and τ_{shift} with system size. Large markers correspond to the results in the main text where we separated retrieval (small seed and $\lambda = 0$) from shift (large seed and $\lambda = 8$). Small markers correspond to the current simulation where seed size and $\lambda = 8$ are fixed. **d**, The fraction of realizations that retrieved the first pattern completely before undergoing any shift, as shown **a**, averaged over 100 realizations. In this analysis we fixed the following simulation parameters $(\mu, \varepsilon, \lambda) = (-20, 11.5.8)$.

1.9 Entropy production

In the equilibrium multifarious assembly regime (Fig. S12**a**), entropy production reflects the transient approach to equilibrium. In the presence of weak non-reciprocal drive, the retrieval period can be observed to be similar to the equilibrium regime (Fig. S12**b**). However, after complete retrieval of the target pattern, $\Delta\Sigma$ continues to increase due to persistent nucleation of small-scale cyclic activities, which are not able to grow to the size of the complete pattern, i.e. they are sub-critical. In the presence of sufficiently strong non-reciprocal drive the probability of shift increases and we observe different scenarios where the initial seed can successfully retrieve the pattern and then undergo shifts (retrieval and shifts), or the small seed undergoes shifts while retrieving the pattern. One can identify two stages of exploration and shift, which can be associated with relatively slow and sharp increases in the entropy production, respectively. Sub-critical nucleation cycles and the corresponding entropy production can also be observed in this regime. Initializing the simulation with the full patterns instead of small seeds (and excluding the retrieval regime) also confirms the above observations as shown in Fig. S13. Very strong non-reciprocal drive leads to the instability of the initial seed, which is similar to melting in equilibrium self-assembly.

As discussed in the main text, entropy production reflects signatures for various dynamical regimes. Figure S13 shows a relatively slow entropy production during relatively stationary states, reflecting on small-scale sub-critical cyclic tile exchanges. Here, the retrieval period is suppressed by choosing the first pattern as the initial seed.

We can also use entropy production to gain more insight into the chimera regime of the state diagram (see Fig. S14). We examine the entropy production in the chimera states corresponding to $\varepsilon \in [24-30]$ in Fig S10**d**. We observe that for weak λ entropy production behaves the same way as in the exploration phase during the retrieval state when λ is weak, namely, continuous entropy production accompanied by no appreciable change in the global pattern. Upon increasing λ, homogeneously-nucleated chimeras ($\varepsilon \in [24-26]$) undergo shift until all parts reach the end of the sequence. A second type of chimeras ($\varepsilon \in [27-30]$) that grow out of a centre seed also start to exhibit the shifting transitions. The central part of the chimera with the initial seed shows exploration-like behaviour in terms of the entropy production. For strong λ, the central part of these chimeras can also exhibit shifts and change to other chimeras that correspond to other seeds at the centre.

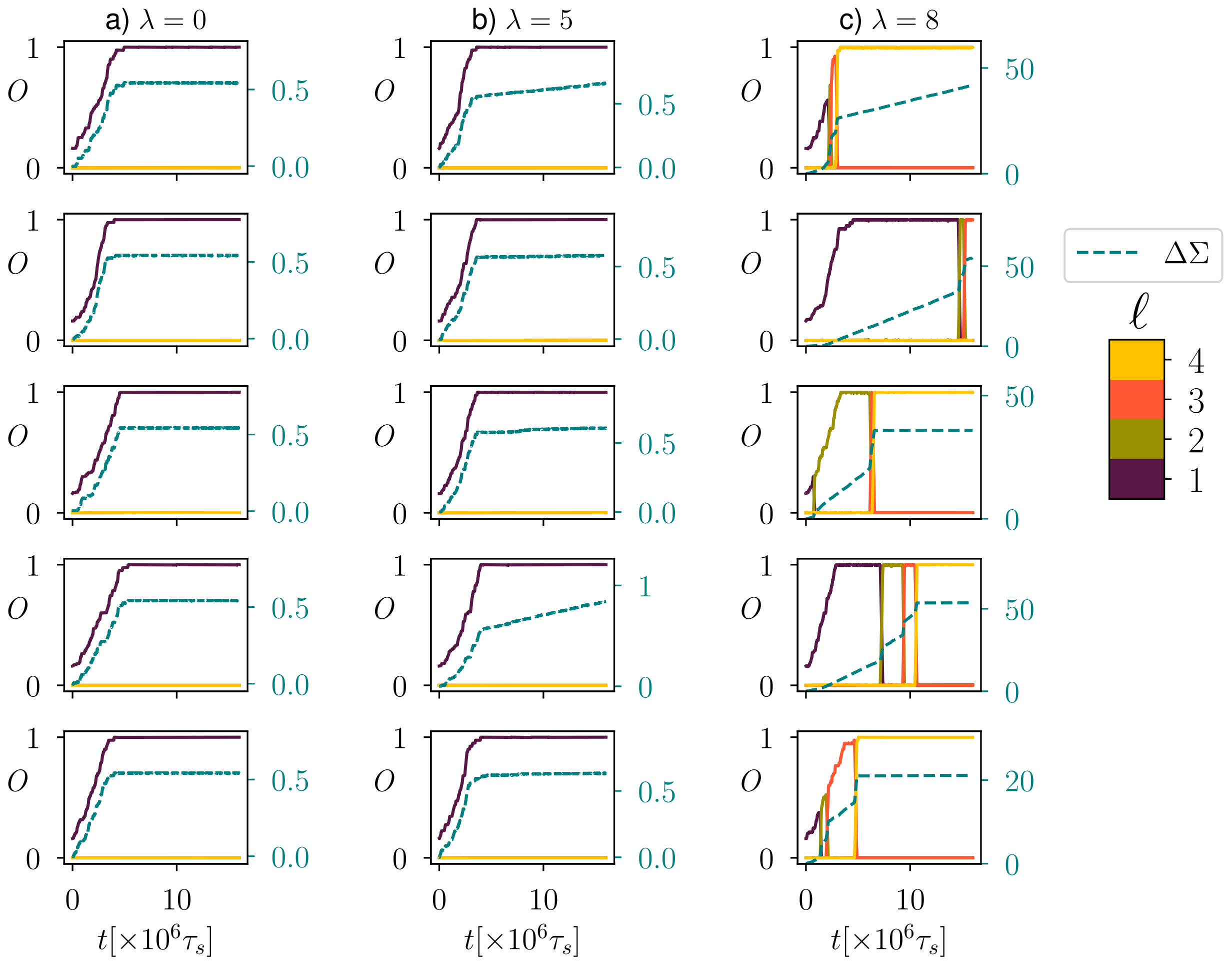

Figure S12: **Entropy production: retrieval versus shift.** Entropy production for equilibrium retrieval in (reciprocal) multifarious-assembly where ($\lambda = 0$) and (non-reciprocal) multifarious self-organization with weak non-reciprocal interactions ($\lambda = 5$) and moderate non-reciprocal interactions ($\lambda = 8$). Four patterns of size 40×40 are stored in the reciprocal interactions of the tiles. In the case of non-zero λ, these four patterns make a sequence of length three; $S^{(1)} \to S^{(2)} \to S^{(3)} \to S^{(4)}$. All simulations start from a small seed of size 16×16 from the first pattern. Each column shows 5 different realizations. Entropy production is calculated as sum of log ratios of probabilities of a move and its reverse and is divided by grid size thereby reporting entropy production per grid point.

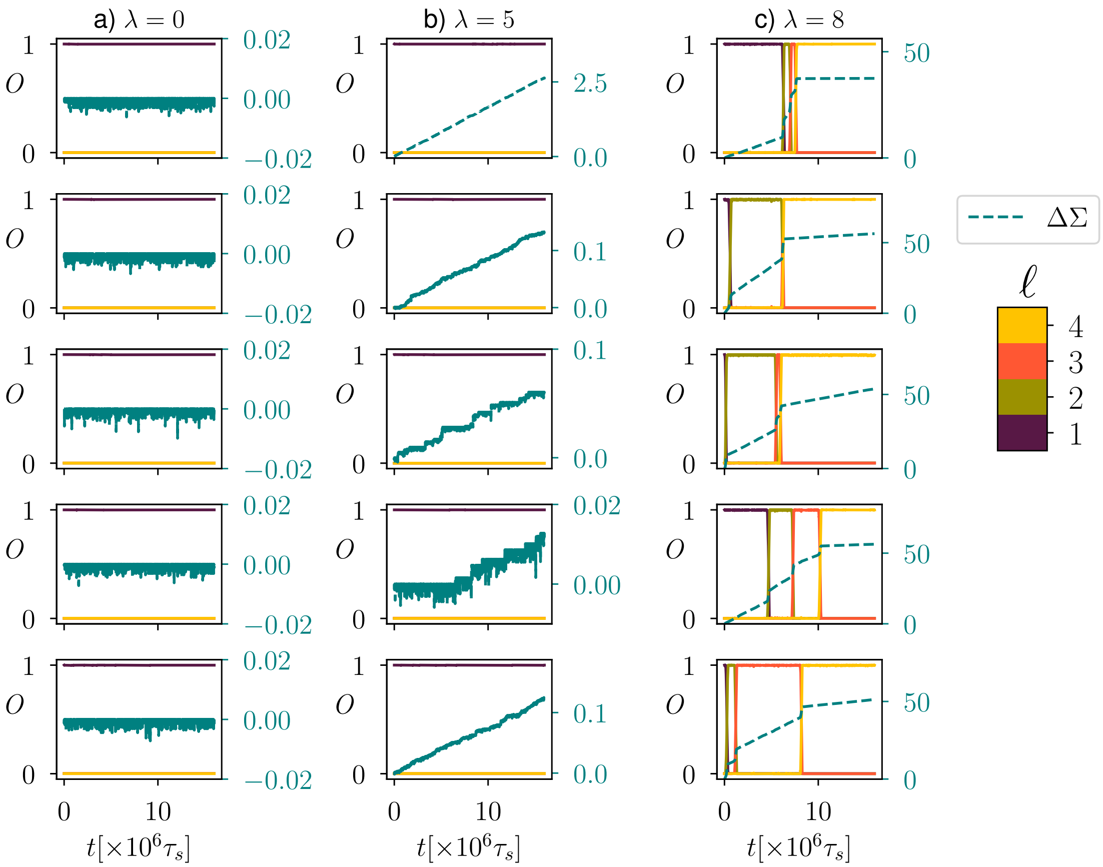

Figure S13: **Entropy production: large seed.** Entropy production in equilibrium retrieval in (reciprocal) multifarious-assembly where ($\lambda = 0$) and (non-reciprocal) multifarious self-organization with weak non-reciprocal interactions ($\lambda = 5$) and sufficiently strong non-reciprocal interactions ($\lambda = 8$). Four patterns of size 40×40 are stored in the reciprocal interactions of the tiles. In the case of non-zero λ these four patterns make a sequence of length three; $S^{(1)} \to S^{(2)} \to S^{(3)} \to S^{(4)}$. All simulations start from the first pattern placed in the middle of the grid as an initial seed. Each column shows 5 different realizations.

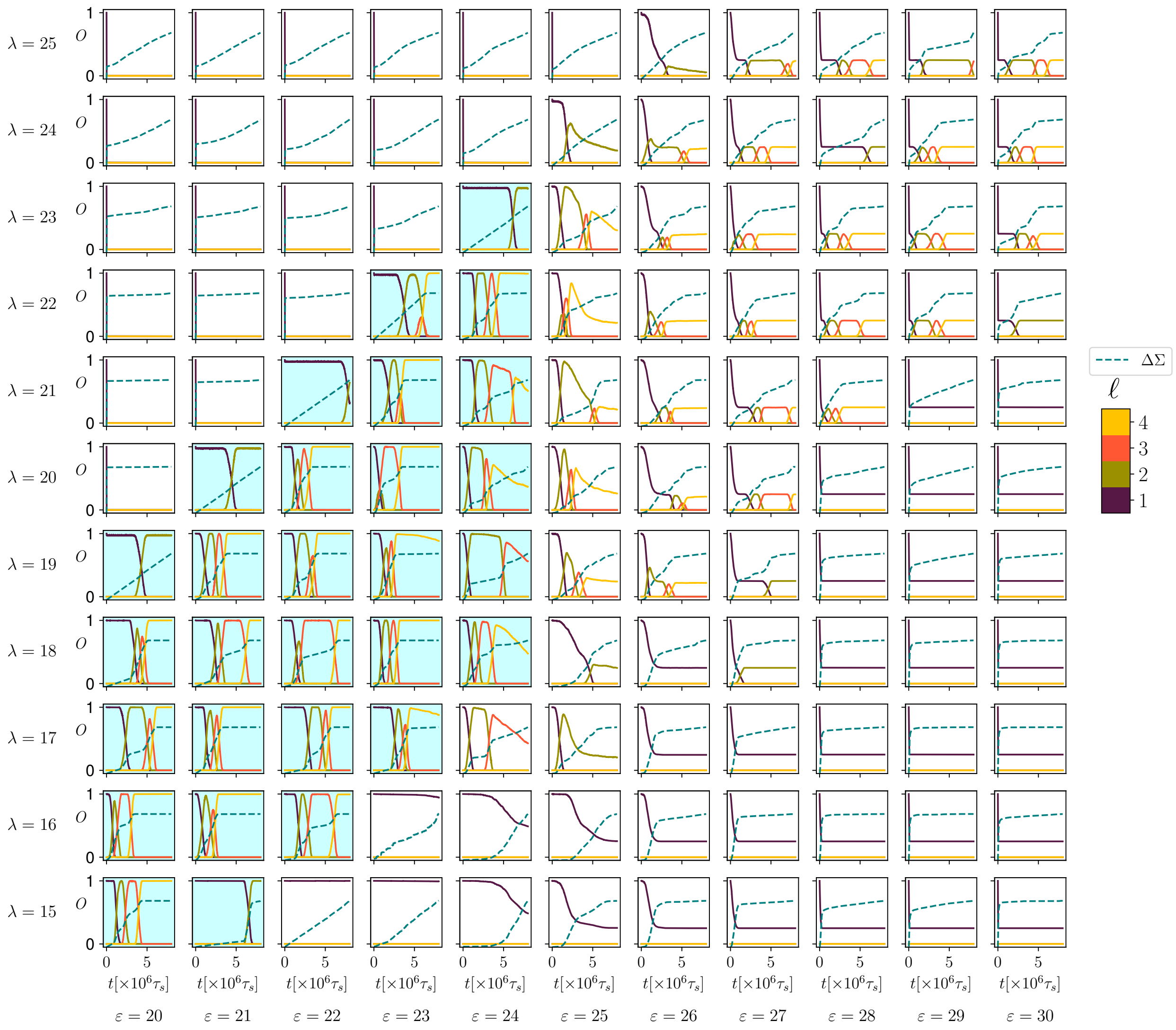

Figure S14: **Entropy production in chimeras.** We plot overlap and entropy production for each pair of (ε, λ) values where $\varepsilon \in [20, 30]$, $\lambda \in [15, 25]$ and $\mu = -30$. Four patterns of size 70×70 are encoded as a sequence of length three; $S^{(1)} \rightarrow S^{(2)} \rightarrow S^{(3)} \rightarrow S^{(4)}$. All simulations start from the first pattern placed in the middle of the grid as an initial seed. Entropy production is plotted versus the right y-axis. Note that chimeras show different behaviour depending on their type (type one for homogeneously-nucleated-like chimeras where $\varepsilon \in [24, 26]$ and type two for chimera with central seed where $\varepsilon \in [27, 30]$), for sufficiently strong λ. The panels with cyan background represent shape-shifting regime that is separated from the chimera regime by type one chimeras.

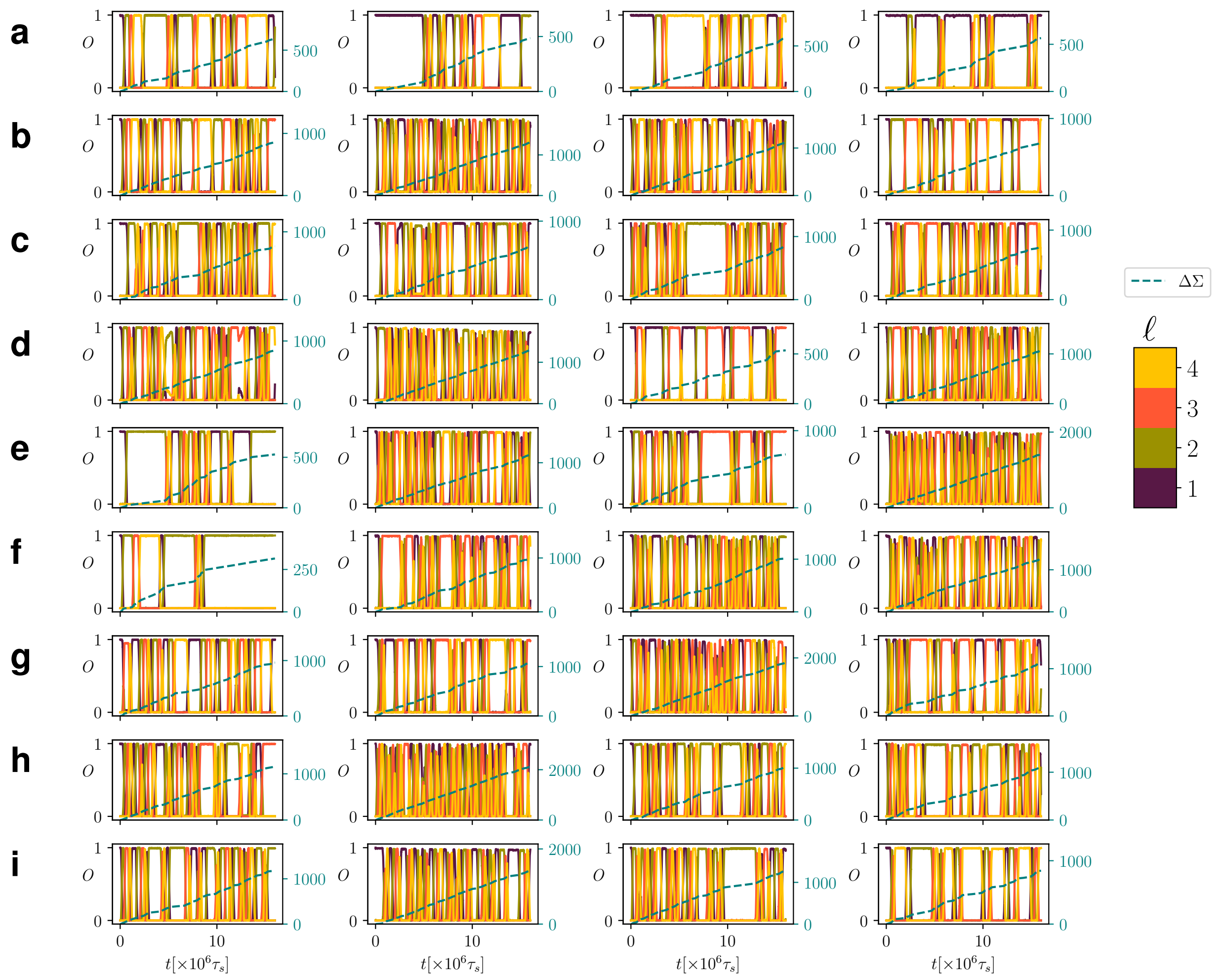

Figure S15: **Cycles of length 4.** Cycles are implemented throughout the multifarious self-assembly region with appropriate selection of λ. Four patterns of size 40×40 are encoded as a cycle of length four; $S^{(1)} \rightarrow S^{(2)} \rightarrow S^{(3)} \rightarrow S^{(4)} \rightarrow S^{(1)}$. Each row corresponds to four different realizations of the loop. Panels **(a)** to **(i)** correspond to the markers listed in Table. 1.

1.10 Cycles

A cycle of shifting patterns can be realized by implementing a closed sequence (loop) in the programming. Here, we show that cycles can be realized robustly within different regions of multifarious-assembly regime with the appropriate values of the strength of non-reciprocal interactions.

Let us implement a cycle of length 4, $S^{(1)} \rightarrow S^{(2)} \rightarrow S^{(3)} \rightarrow S^{(4)} \rightarrow S^{(1)}$ of 4 patterns of size 40×40. For 9 different points in the multifarious-assembly regime and different values of λ for each pair of (μ, ε), we show four different realizations in Fig. S15. We observe that cycles can be realized robustly in any region of multifarious-assembly regime. Entropy production highlights the exploration and shift behaviour in all the cases. One can initiate a cycle starting from any pattern in the sequence and obtain the sequence in the correct order, as shown in Fig. S16.

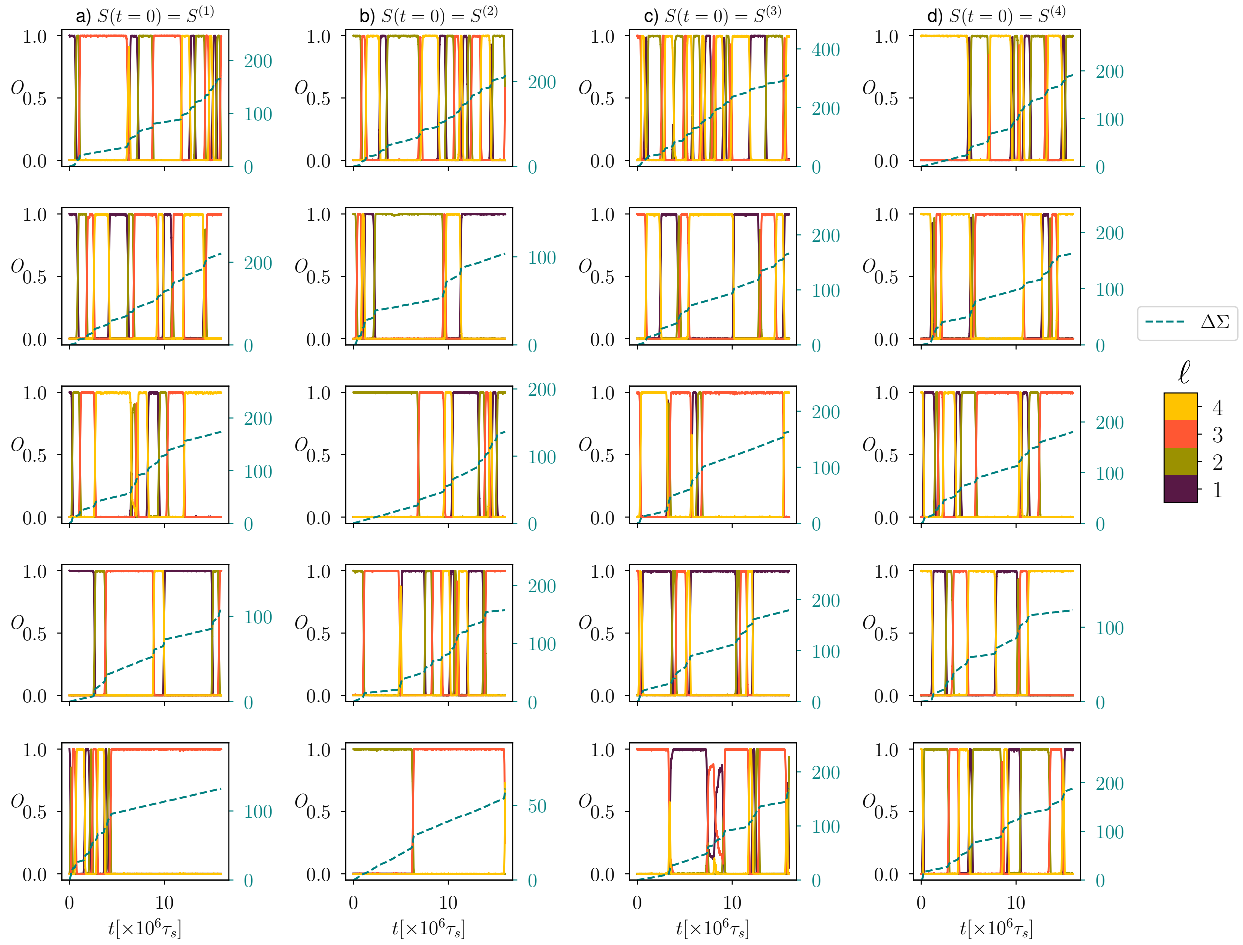

Figure S16: **Initialization of cycles.** A cycle can be initialized from any of the structures in the sequence. Four patterns of size 40×40 are encoded as a cycle of length four; $S^{(1)} \to S^{(2)} \to S^{(3)} \to S^{(4)} \to S^{(1)}$. Each column corresponds to five different realizations of the cycles. Panels **(a)**-**(d)** correspond to the realizations that start with $S^{(1}$-$S^{(4)})$ as initial seed. The following parameters are used in the simulations $(\mu, \varepsilon, \lambda) = (-20, 11.5, 8)$.

1.11 Period of cycles

We have shown that cycles are realizable throughout the multifarious-assembly regime. Here, we show that λ plays the role of a control knob to set the time scale of cycles or their period. For small values of λ, shifts happen rarely and there is no interference between different shifts, and a long simulation time will be needed in this case in order to observe several instances. On the other hand, for large values of λ shifts and premature shifts can happen frequently. Figure S17 shows the behaviour of the cycle period τ_{cycle} as a function of λ. Upon increasing λ beyond the minimum threshold, the period becomes more or less independent of λ, as shown in Fig. S17**a**. Very close to $\lambda_{\max}$, there is an increase in τ_{cycle}. This is due to the instability effect, and the realizations in those regions are accompanied by some errors, as shown in Fig. S17**j**.

1.12 Basins of attraction for cycles

Here, we present the results of analysis on the time scale of the short loops of length three, four and five and visualize the basins of attraction in the case of the cycles of length three.

We define τ_{cycle} as the time that the system has strong overlap with one of the structures in the sequence, divided by the number of structures realized during this time; i.e., it represents residence time per structure in a cycle. At $\lambda_{\min}$, the system is occupied with only one pattern (initial seed) for the whole simulation time, and since no shift is realized, τ_{cycle} is the simulation time. At $\lambda_{\max}$, the initial seed is highly unstable and immediately melts. In this case only a single pattern appears (initial seed) and then melts in a short time, which identifies τ_{cycle} as the melting time. In between, we have stable cycle with near constant τ_{cycle}; see Fig. S18**a**. Close to the instability ($\lambda_{\max}$) τ_{cycle} starts to increase. In this case, we observe quasi-stable shifts as shown in Fig. S17. The transition points $\lambda_{\min}$ and $\lambda_{\max}$ can be determined by fluctuations of τ_{cycle} defined as $\chi = \langle \Delta\tau_{\text{cycle}} \rangle \,/\, \langle \tau_{\text{cycle}} \rangle$ (see Figs. S18**a**-**c**). In comparison to the loose bounds on λ defined above, the two peaks in χ capture the bounds more accurately. For a cycle of length 3 more systematic analysis can be done by looking at the low dimensional space of overlap; $\{O_1, O_2, O_3\}$. Example of cycles in this space are shown in Fig. S18**d**. For moderate values of λ as shown, the trajectory can undergo many shifts, as well as premature shifts due to fluctuations.

We would like to highlight a remarkable emergent feature of our non-reciprocal model: the ability to push the system out of kinetic traps. To address this feature, we consider formation of chimeras as a typical kinetic trap in self-assembly. In this case, while system tries to assemble the desired structure, it may be trapped in the undesired chimeras due to the promiscuity of the tiles. To illustrate this, we design the following *in silico* experiment. We store three structures in the pool and examine the low dimensional dynamics of the system in O-space. In the triangular space of overlaps, the main desired structures reside on the vertices. Within the triangle we intentionally create chimeras that are mixtures of different structures depending on how close are they to each of the vertices as shown in Fig. S18**e** at $t = 0$. We then simulate the system for a long time ($t = 10^7 \tau_s$ steps) and record the error. In the equilibrium case, as time goes on the chimeras that are close to the desired structures are absorbed to these basins of attraction. However the strong chimeras in the middle cannot leave these states, as they are kinetically trapped, and it takes a very long time for them to correct the errors and leave these traps; see Figs. S18**e** and **f**. We redo the same simulation with $\lambda = 16$. The non-equilibrium dynamics pushes all the systems towards shifts by exploiting the non-equilibrium drive provided by non-reciprocal interactions, hence efficiently converging to the cycle regime, as shown in Figs. S18**g** and **h**.

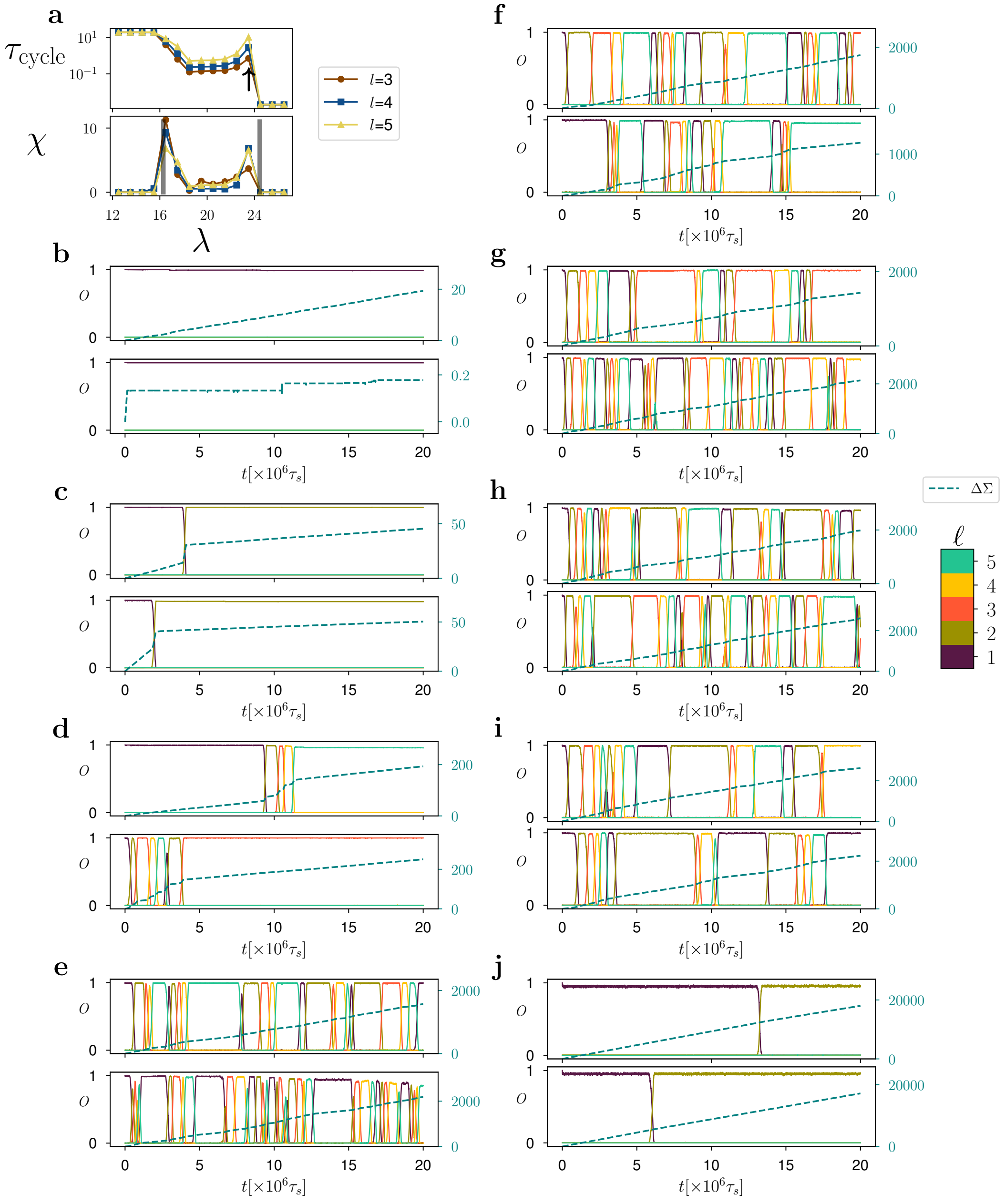

Figure S17: **Time scale of cycles.** **a**, τ_{cycle} as defined in the text for cycles made of 3-5 structures and different value of λ (top), and its fluctuations (bottom). Each marker is averaged over 100 independent simulation. Vertical grey lines show the loose bounds and the arrow shows the approach to instability corresponding to **j**. Panels **b**-**j** are two realizations for each marker in the grey window of **a**. The simulation were initialized with the first pattern and continued for $20 \times 10^6 \tau_s$ steps.

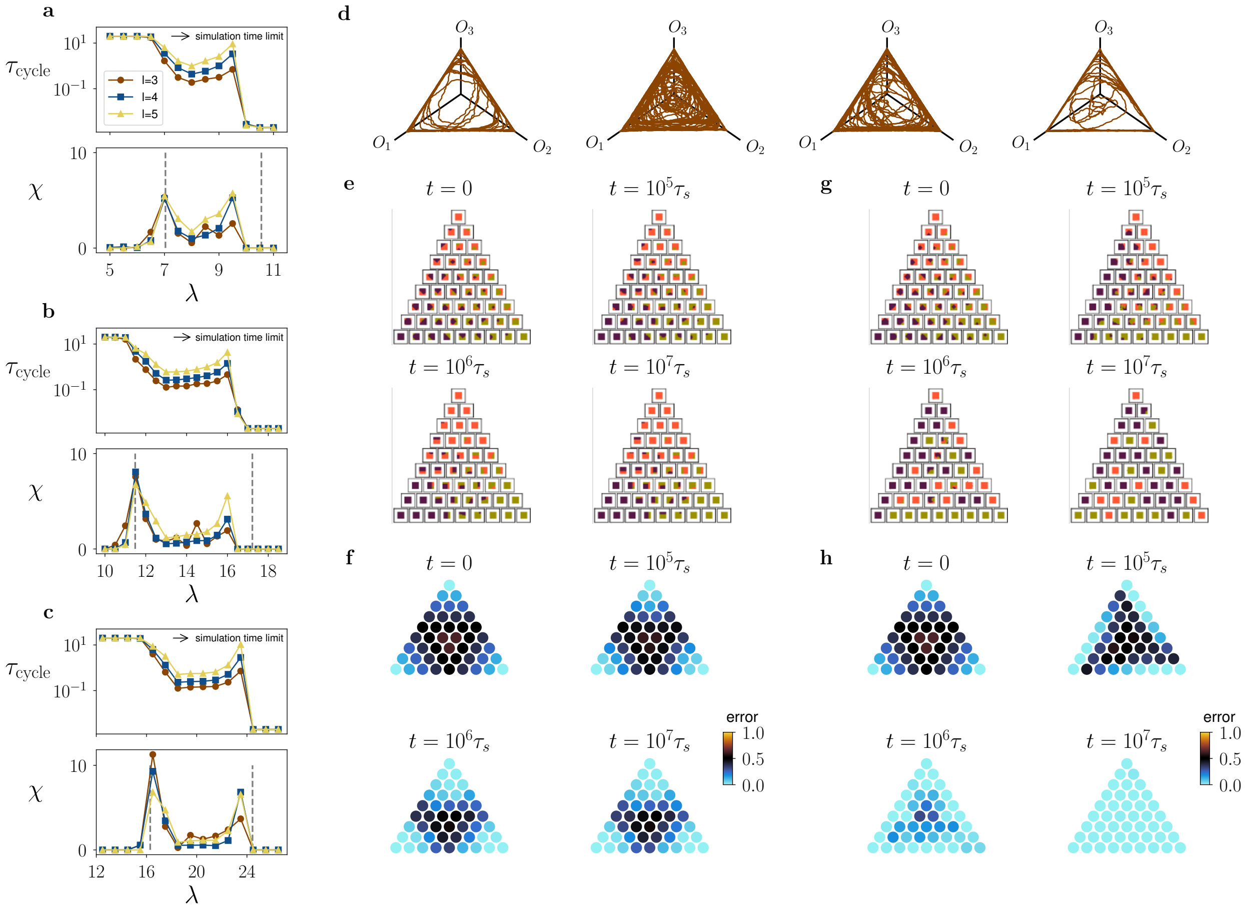

Figure S18: **Basins of attraction for cycles.** **a**, The average duration of a realized structure in implementation of loops of length three, four and five (top). The relative fluctuations $\chi = \langle \Delta\tau_{\rm cycle}\rangle / \langle \tau_{\rm cycle}\rangle$ (bottom). The two peaks roughly correspond to the values of $\lambda_{\rm min}$ and $\lambda_{\rm max}$ (dashed lines correspond to the loose bounds $\lambda_{\rm min} \approx \frac{2}{3}\varepsilon$ and $\lambda_{\rm max} \approx \varepsilon$). Panels **a-c** have (λ, ε) coordinates that correspond to $\square, \triangleleft, \diamond$ markers in Table 1. **d**, Four different realizations of cycles of length three with $\lambda = 20.5$ and (μ, ε) corresponding to $\diamond$, in the low dimensional space of $\{O_1, O_2, O_3\}$. **e**, At $t = 0$, the system is initiated with chimeras made of three structures on the vertices of the triangle and a smooth interpolation between them. Deep in the multifarious regime ($\diamond$) and starting from this initial state, evolution of the system is shown at different times for the equilibrium case where $\lambda = 0$. **f**, The average error where each point is averaged over 10 different realizations of chimera. Panels **g** and **h** are the same as **e** and **f**, but for the non-reciprocal case with $\lambda = 16$. Non-reciprocal interactions help the system escape chimeric kinetic traps.

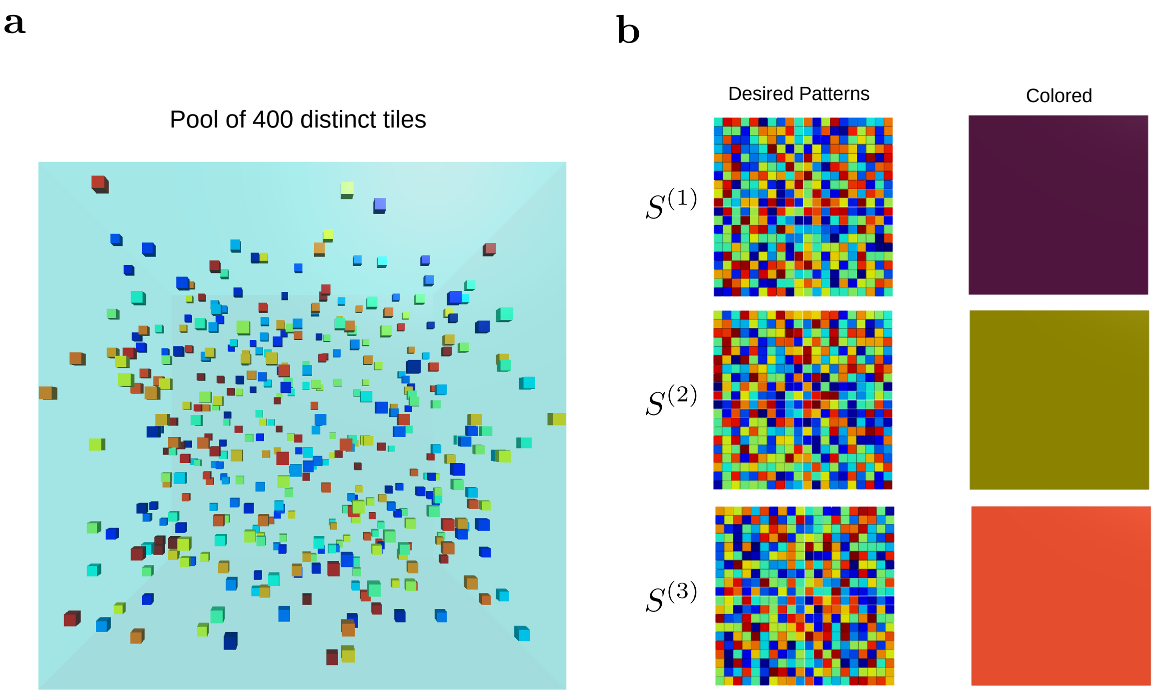

Figure S19: **Brownian dynamics simulation: basic setup.** Panel **a** shows a pool of 400 distinct tiles. **b**, 3 desired 2D structures of size 20×20 made from the 400 tiles. Desired structures are encoded in the reciprocal specific interactions between the tiles as explained in the main text. Side lengths of the simulation box $\sqrt{N}$ is proportional to side length of the structure; $(\sqrt{N_x}, \sqrt{N_y}, \sqrt{N_z}) = (2 \times \sqrt{M}, 2 \times \sqrt{M}, 2 \times \sqrt{M} + 1)$. In all the simulations, the initial seed is placed at $z_{\text{target}} = \sqrt{M} + 1$.

2 Brownian Dynamics Simulation

Here we present a more realistic implementation of the non-reciprocal multifarious model, which provides hints towards experimental realizations of shape-shifters. We designed a stochastic dynamics model of tiles with directional interactions in 3D as shown in S19. There is a region with the same size as the target structures in the middle of the box, which we call the region of interaction. We place the initial seed in this region and let it interact with random tiles reaching to the surface of the structure at $z = z_{\text{target}} \pm 1$ through Brownian motion.

Random movement of a tile depends on its distance from the initial seed or the interaction region introduced in the middle of the box at z_{target}:

- If the tile is exactly placed in the interaction region it does not move.
- If the tile is away from the interaction region, $z > z_{\text{target}} + 1$ or $z < z_{\text{target}} - 1$, then it can diffuse in the discrete space via discrete Brownian dynamics. Each of the 6 directions is chosen with $p = 1/6$, and the move is performed only if the hosting grid point is empty.
- If the tile is within the interaction region at $z = z_{\text{target}} + 1$ ($z = z_{\text{target}} - 1$) then it can interact

with the tile below (above) it and push it out of the trap, provided there is an empty grid point, in order to respect the excluded volume effect. Then, the new tile replaces the old tile. An arriving tile is considered for this kind of interaction if it makes at least one reciprocal or non-reciprocal interaction with one of the neighbours of the existing tile. Otherwise, it undergoes random diffusion. This condition enables the growth of a new pattern within the old one. A replacement of the tile happens with a rate that is proportional to $p = \min\{1, e^{\Lambda-\Delta\mathcal{H}}\}$, where $\mathcal{H}$ now includes only the energy due to specific interactions between the tiles.

The simulation starts with n_{copies} of each tile in the box and the initial seed located at z_{target}. The random walk of the tiles is performed in discrete time and discrete space with periodic boundary conditions. At each time step all the tiles are moved.The simulation continues for $\mathcal{T}$ time steps. During the simulation, the location of each tile is recorded and the error is calculated with the tiles located in the interaction region. Figure S20 shows an example of the time evolution of such a simulation, which features premature shifts.

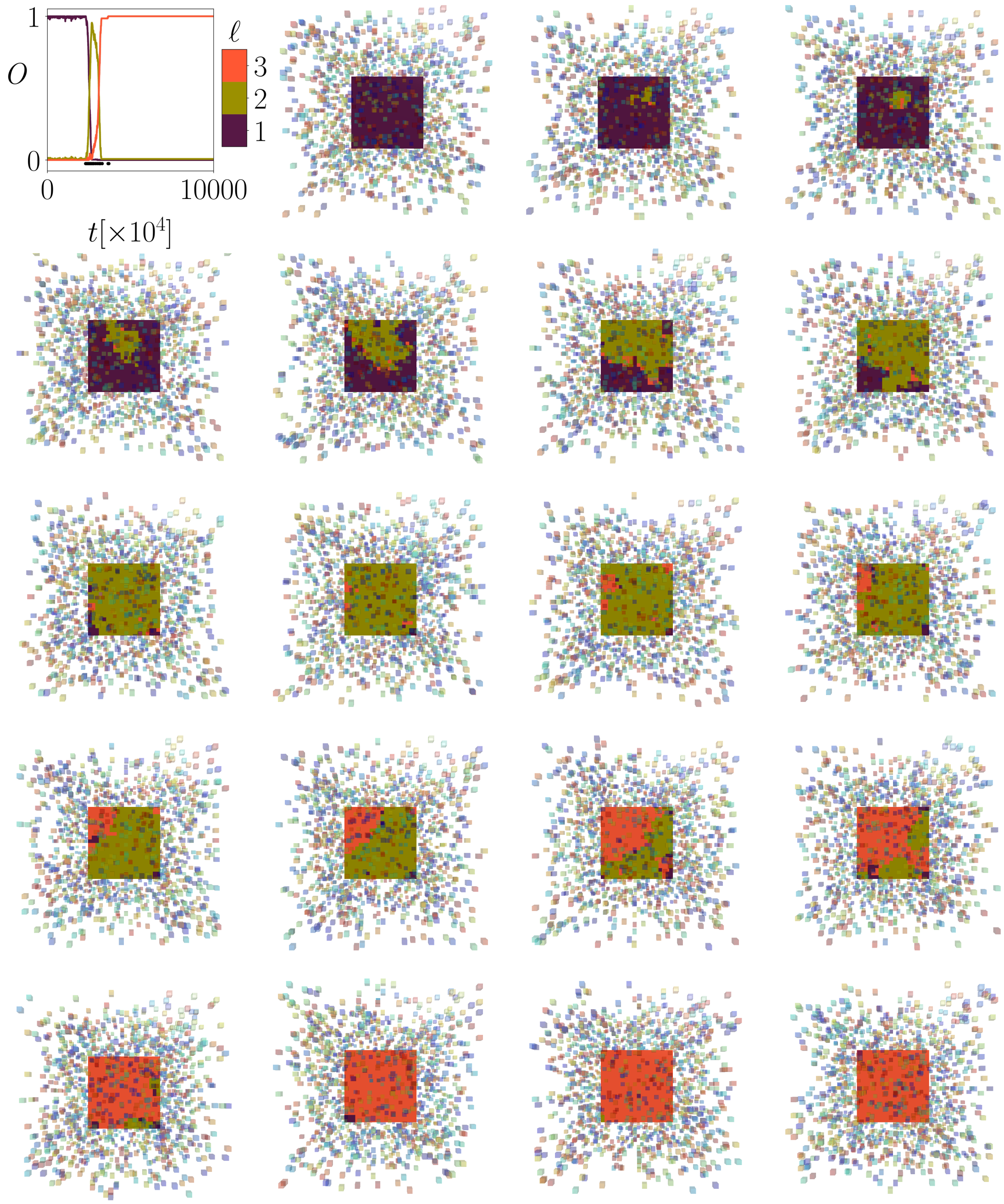

Figure S20: **Brownian dynamics simulation: premature shifts.** **a**, The evolution of the overlap of the part of the system that contains the initial seed with the desired patterns. Patterns are 20×20 structures made of 400 distinct tiles. Beside the initial seed the box contains four more copies of each tile, i.e., in total 2000 tiles are used in simulation. **b**, Snapshots of the system taken from the time steps marked with black dots in **a**. Other simulation parameters: $(\varepsilon, \lambda) = (11.5, 8.0)$. Notice the occurrence of premature shifts.
